# Retinal hyperspectral imaging in mouse models of Parkinson’s disease and healthy aging

**DOI:** 10.1038/s41598-024-66284-7

**Published:** 2024-07-12

**Authors:** Paul Trlin, Jenny Gong, Katie K. N. Tran, Vickie H. Y. Wong, Pei Ying Lee, Anh Hoang, Da Zhao, Leah C. Beauchamp, Jeremiah K. H. Lim, Andrew Metha, Kevin J. Barnham, David I. Finkelstein, Bang V. Bui, Phillip Bedggood, Christine T. O. Nguyen

**Affiliations:** 1 Department of Optometry and Vision Sciences, University of Melbourne, Parkville, VIC 3010 Australia; 2 Florey Institute of Neuroscience and Mental Health, Parkville, VIC 3010 Australia; 3 Present Address: Ann Romney Center for Neurologic Diseases, Brigham and Women’s Hospital and Harvard Medical School, Boston, MA 02115 USA; 4 Present Address: Discipline of Optometry, School of Allied Health, University of Western Australia, Crawley, WA 6009 Australia

**Keywords:** Hyperspectral, Parkinson’s disease, A53T, TauKO, Ageing, Alzheimer’s disease, Neuroscience, Biomarkers

## Abstract

Retinal hyperspectral imaging (HSI) is a non-invasive in vivo approach that has shown promise in Alzheimer’s disease. Parkinson’s disease is another neurodegenerative disease where brain pathobiology such as alpha-synuclein and iron overaccumulation have been implicated in the retina. However, it remains unknown whether HSI is altered in in vivo models of Parkinson’s disease, whether it differs from healthy aging, and the mechanisms which drive these changes. To address this, we conducted HSI in two mouse models of Parkinson’s disease across different ages; an alpha-synuclein overaccumulation model (hA53T transgenic line M83, A53T) and an iron deposition model (Tau knock out, TauKO). In comparison to wild-type littermates the A53T and TauKO mice both demonstrated increased reflectivity at short wavelengths ~ 450 to 600 nm. In contrast, healthy aging in three background strains exhibited the opposite effect, a decreased reflectance in the short wavelength spectrum. We also demonstrate that the Parkinson’s hyperspectral signature is similar to that from an Alzheimer’s disease model, 5xFAD mice. Multivariate analyses of HSI were significant when plotted against age. Moreover, when alpha-synuclein, iron or retinal nerve fibre layer thickness were added as a cofactor this improved the R^2^ values of the correlations in certain groups. This study demonstrates an in vivo hyperspectral signature in Parkinson’s disease that is consistent in two mouse models and is distinct from healthy aging. There is also a suggestion that factors including retinal deposition of alpha-synuclein and iron may play a role in driving the Parkinson’s disease hyperspectral profile and retinal nerve fibre layer thickness in advanced aging. These findings suggest that HSI may be a promising translation tool in Parkinson’s disease.

## Introduction

Hyperspectral imaging (HSI) combines imaging and spectroscopy to capture both spatial and spectral tissue information. By projecting a light source of varying wavelength onto the retina and detecting differences in the intensity of reflected light, the influence of different materials and proteinopathies on light-tissue interactions can be explored^1^. Significant differences in reflectance at key wavelengths throughout the spectrum can identify tissue properties and inform diagnosis and understanding of disease progression^2^, providing a non-invasive alternative to existing imaging and diagnostic techniques. As such the retina has the potential to provide a cheaper, more accessible and less invasive alternative to brain imaging (i.e. Positron emission tomography, PET) or cerebrospinal fluid approaches that require lumbar puncture^3,4^. HSI images are fast to acquire, well tolerated and repeatable, making HSI suitable for broad population screening.

Our work and others have reported hyperspectral changes in Alzheimer’s disease (AD). More specifically, HSI of the retina has been applied to AD ex vivo tissue^5^, animal models^6–8^ and human patients with and without amyloid-beta load^6^. To our knowledge, no one has yet applied HSI to human or animal models of Parkinson’s disease (PD). The pathological hallmarks of PD include abnormal alpha-synuclein (α-synuclein) protein accumulation and iron accumulation in the brain. Increased levels of α-synuclein in the retina have also been found in both patients with PD^9^ and animal models of PD^9,10^. Like the substantia nigra region of the brain, the outer retina and retinal pigment epithelium (RPE) are naturally rich in iron. Baksi and Singh^11^ demonstrate in both cell culture and in an in vivo animal model that the presence of α-synuclein in the retina causes over-accumulation of iron-rich ferritin. As such, imaging the retina targeting α-synuclein or iron pathways may be useful for detecting PD. Interestingly, some studies have examined other spectral techniques applied to in vitro and ex vivo preparations of PD that target these pathogenic pathways.

Mammadova et al.^10^ examined retinal cross sections from the A53T mouse model of α-synuclein deposition using Raman spectroscopy. They found that by employing a single excitation wavelength (532 nm), emission across 20 spectra A53T retina could be discriminated from controls. Similarly, Fazili et al.^12^, also employed a monochromatic excitation wavelength (350 nm) on in vitro preparations of native α-synuclein and observed increased Rayleigh scatter within the emission band across 300 to 400 nm^12^. Although these approaches did not use multiple wavelengths (i.e. were not hyperspectral) they illustrate that α-synuclein promotes light scatter when lower wavelengths within the UV/visible light spectrum are used. Furthermore, Oh et al.^13^ found that when HSI was applied to isolated iron (ferric ammonium citrate), increased reflectance was observed in the visible light spectrum. Moreover, in areas of high iron density in a cell culture model they found reflectance was altered mainly within a ~ 475 to 620 nm band (based on concentration of iron estimate to return 50% of maximal reflectance, i.e. EC_50_ estimate). These results show promising findings of detecting features of α-synuclein accumulation or iron overload using different wavelengths of light in in vitro models. Our study will extend this to examine HSI reflectance in retinae of PD mouse models.

It is established that the retinal nerve fiber layer (RNFL) layer is reflectile in the blue short-wavelength spectrum^14–16^ and meta-analyses show patients with PD exhibit RNFL thinning^17,18^. As such, it is possible that RNFL thinning may alter HSI at shorter wavelengths. Moreover, even healthy aging in the absence of disease is known to exhibit RNFL thinning and thus comparing healthy age-related changes in HSI to age-related neurodegenerative diseases is key.

The A53T mouse model overexpresses human α-synuclein which has been found in the brain^19–22^ and the retina^10,23^. Motor deficits characteristic in PD are also well characterized^19–22^. Iron overload has been observed in the Tau knock out (TauKO) mouse model^24–27^ as well as alteration to other sensory systems^24,28^. The current study aims to examine HSI in these two animal models (A53T and TauKO) at various disease stages and correlate this with retinal tissue levels of α-synuclein and iron. HSI in PD models will also be compared with the effects of normal healthy aging.

## Methods

### Animal models

#### Animal ethics and husbandry

All experimental procedures involving animals were performed with approval from the Howard Florey Institute Animal Ethics Committee (Approval Numbers: 17-046-UM, 18-072-UM, 21-019-UM) and in accordance with the ARRIVE guidelines. In total *n* = 105 A53T, *n* = 52 TauKO, and *n* = 26 C57blk6/J mice were bred and housed at the Melbourne Brain Centre’s animal facility in well-ventilated cages (Green IVC Sealsafe Plus Mouse, Techniplast, Buguggiate, VA, Italy), where an ambient temperature of 20 °C was maintained and a 12 h light–dark cycle was used. Water and Food (Barastoc mouse pellets, Ridley Corporation, Melbourne, VIC, Australia) were provided ad libitum.

#### A53T mice

The hA53T transgenic line M83 (A53T, JAX stock #004479; B6;C3-Tg(Prnp-SNCA*A53T)83Vle/J) was used in this study due to its widespread expression of mutant human A53T α-synuclein translocation (8–12 months) which parallels the onset of its well characterized PD phenotype. Mice were genotyped with two probes to confirm hA53T homozygosity (SNCA-2 Tg and Chr12-3 WT, Transnetyx, Cordova, TN, USA). As the background strain of these mice (B6C3H) expresses the Pde6b^rd1^ allele which leads to retinal degeneration, mice were also genotyped for the Pde6b^rd1^ gene (Transnetyx, Cordova, TN, United States) and only mice without this gene were used.

Hyperspectral imaging was performed in 4-month-old (*n* = 15), 6-month-old (*n* = 17) and 14-month-old (A53T, *n* = 18) mice and wild-type (WT) counterpart (4-month: *n* = 15, 6-month: *n* = 15, 14-month: *n* = 15) cohorts. These ages were chosen because they have been shown to characterize the prodromal, early/mid and late disease stages well^10,21,23^.

#### TauKO mice

The Sv129B/6 tau^−/−^ mice also known as Tau knock-out (TauKO) mice were used to represent a model completely ablated of tau protein. These mice were bred in-house along with their wild type controls (Sv129B/6 tau^+/+^). Tau pathology leads to a degenerative phenotype with advanced aging, mirroring the general trajectory of PD. However, complete ablation of tau proteins in previous mice models have shown to impair age-dependent motor development therefore making tau a biomarker of interest for PD^25–27^.

Beauchamp et al.^28^ have shown that 7-month-old age group represents a prodromal PD timepoint where non-motor olfactory changes have been found before the progression of motor symptoms which manifest by 15-months; consistent with typical age associated disease progression for PD^29^. As such 8-month-old mice were imaged (TauKO: *n* = 13 vs. WT *n* = 10) to characterize the changes in the early phases of disease progression to the more advanced stages (18-months-old age group, TauKO: *n* = 16 vs. WT: *n* = 14).

#### 5xFAD mice

As previously described by our group the 5xFAD mouse is a useful AD model to examine the hyperspectral phenotype^6,7^. It has 5 familial human AD genes and a C57blk6J background which does not contain the Pde6bdr1 gene for retinal degeneration. It exhibits retinal amyloid-beta accumulation^30^ and shows hyperspectral changes at 6–18 months of age. The current study examined 12-month-old 5xFAD mice (*n* = 9) compared with age-matched WT littermates (*n* = 9) to compare against the PD animal models.

#### Healthy aging

To examine whether advancing age influenced HSI measures, WT littermates of the A53T and TauKO cohorts were compared between across age. The TauKO littermates, i.e. Sv129B/6 mouse strain, were compared between 8- and 18-months of age (*n* = 10, *n* = 14, respectively). The A53T WT littermates, i.e. B6C3H mice, were compared between 6- and 14-months of age (*n* = 15, *n* = 10, respectively). Note that the 4-month-old A53T WT littermates were not used in this comparison as there was a modification in set-up (angular orientation of a pellicle beam-splitter) at this age, causing a shift in the raw spectrum due to the angular dependence of thin film reflection. However, comparison between 4-month-old A53T and 4-month-old WT littermates is still warranted given they were collected under the same conditions (matching pellicle orientation). To remove any bias associated with different pellicle corrections between groups, we included the non-pellicle corrected data and avoided direct comparisons between groups where the angular orientation of the pellicle changed. As an independent comparator for young, mid-age and older-age a separate cohort of C57BL/6 J mice were also examined at 3 key time points (3-months: *n* = 9; 6-months: *n* = 9; 12-months: *n* = 8).

#### General procedures

Imaging was conducted under general anesthesia of 80:10 mg/kg Ketamine:Xylazine (Ilium Ketamil 100 mg/ml and Ilium Xylazil 100 mg/ml, Troy Laboratories Pty Ltd, Smithfield, NSW, Australia). Topical aaesthetic (0.5% proxymetacaine, Alcaine™, Alcon Laboratories, Frenchs Forest, NSW, Australia) and mydriatic eyedrops (1% tropicamide, Mydriacyl, Alcon Laboratories, Frenchs Forest, NSW, Australia) were instilled. For older mice who experience poorer dilation (14-month-old A53T and 17-month-old TauKO) an additional mydriatic drop of phenylephrine 2.5% was applied. During anesthesia, body temperature was maintained at 37 °C using a heat pad. Corneal hydration was maintained using an ocular gel (GenTeal, Novartis Pharmaceuticals Pty Ltd, Macquarie Park, NSW, Australia) under a glass coverslip (Knittel Glass, Bielefeld, Germany).

### Hyperspectral imaging

As previously detailed in Lim et al.^7^, the HSI platform was a custom-built bench ophthalmoscope that consists of a fast-switching monochromator light-source (Polychrome V, Till Photonics, Hillsboro, OR, USA), which directed light to a semi-reflective pellicle beam splitter (BP245B1, ThorLabs, Newton, NJ, USA) and then into the eye. Fourty-five percent of incident light was directed into the rodent eye via the pellicle beam splitter which is then returned from the retina through diffuse reflection and passed through the pellicle again. Hyperspectral images are influenced by the light output, as well as the pellicle reflectance and transmittance, which can create a sinusoidal artefact^7^. Light reflected from the animal’s eye is transmitted through the beam splitter and focused by a series of lenses onto the “Neo” scientific complementary metal oxide semiconductor (sCMOS) camera (Andor Technology, Belfast, UK). Anesthetised animals were placed on a 3-dimentional cradle platform (ThorLabs, Newton, NJ, United States) in front of the pellicle. Images were taken with a 500 × 500 pixel region of interest centred on the optic nerve (Metamorph Software, Molecular Devices, San Jose CA, USA). Image acquisition was performed in a sequence from 320 to 680 nm (1 nm increments each with a 10 nm bandwidth) to capture a stacked image (TIFF format) where each slice represents one wavelength of incident light. Each wavelength takes 100 ms to cycle through, allowing the hyperspectral system to sequence 361 wavelengths in 36.1 s (Supplementary Fig. 1).

#### HSI processing and analysis

**Figure 1:**
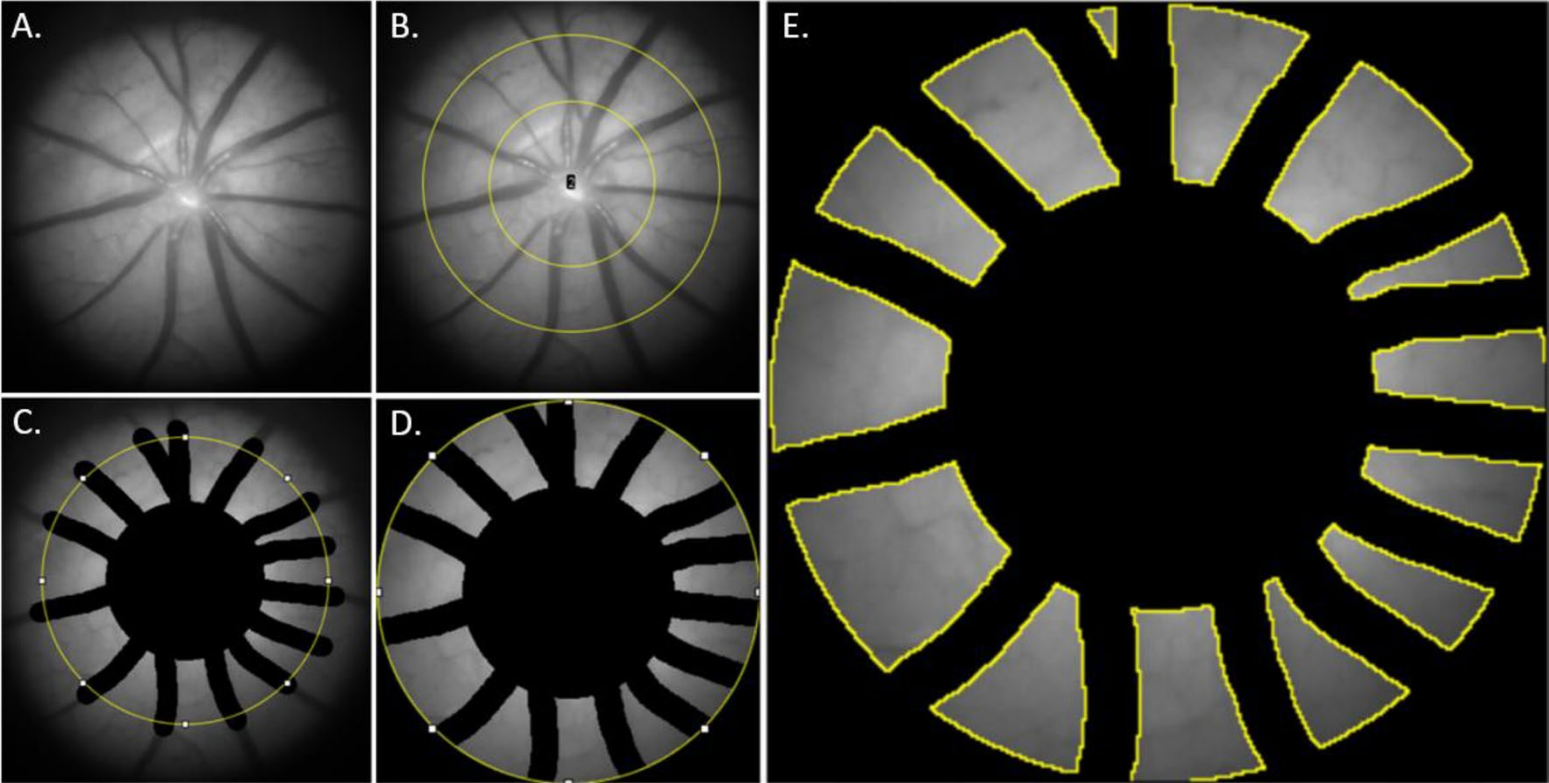
Image analysis masking process (**A**) Retinal image at 427 nm selected where blood vessels are visible. (**B**) Application of an optic nerve and pupil annulus with a fixed size are applied. (**C**) Vessels, optic nerve head and peripapillary region within the annulus centre are masked (**D**) Area outside of pupil annulus is masked (**E**) The remaining retinal regions of interest (within yellow borders) were analysed for reflectance.

Images from each region were exported as 16-bit greyscale TIFF images, with each stack including 361 slices representing different wavelengths of incident light from 320 to 680 nm. All images were analyzed using an open-source FIJI software package^31^ to collect median reflectance data from the same region of interests across all 361 slices. In FIJI, to compensate for eye/breathing movements each slice was registered to frame 108 (427 nm) where there is a high contrast between the blood vessels and the retina using the built-in algorithm “StackReg(Translation)”^32^ (Fig. 1A). Briefly, this method of image registration utilises a sub-pixel algorithm that minimizes the mean square difference of intensities between the reference image and subsequent images in the stack^32^. The ‘translation’ method used constrains the motion correction to lateral movement (x, y) without rotating, resizing the image or changing pixel intensity*.* A fixed retinal region of interest (ROI, Fig. 1E) was selected by manually masking the optic nerve head (159 pixel diameter, Fig. 1B), masking the smallest unvignetted field of view across all animals (286 pixels diameter, Fig. 1B) to ensure an area of even illumination was analyzed, and masking the major blood vessels (Fig. 1C, 20-pixel thickness). The image was then cropped to minimise file size and improve processing speed (Fig. 1D).

Median reflectance data were collected from the retinal ROI for each of the 361 slices using the built-in Measurestack macro^31^. Data were then down-sampled in 5 nm steps (Microsoft Excel, Microsoft) by returning every 5th wavelength, i.e. 320, 325, 330 nm etc. To correct for overall differences in luminance, reflectance profiles were “baseline corrected” by subtracting the average reflectance from 320 to 360 nm (Eq. 1, UV wavelengths where spectral sensitivity of the camera should be negligible) and normalized to the average reflectance at a long wavelength band (630–660 nm, Eq. 2) consistent with previous studies^5,7,8^.

1 $$Baseline\, corrected \,reflectance_{{\lambda_{n} }} = reflectance_{{\lambda_{n} }} - Average\, reflectance_{{\lambda_{{320 - 360\,{\text{nm}}}} }}$$

2 $$Normalised \,reflectance_{{\lambda_{n} }} = \left( {\frac{{Baseline\, corrected\, reflectance_{{\lambda_{n} }} }}{{Average \,reflectance_{{\lambda_{{630 - 660\,{\text{nm}}}} }} }}} \right) \times 100$$

**Figure 2:**
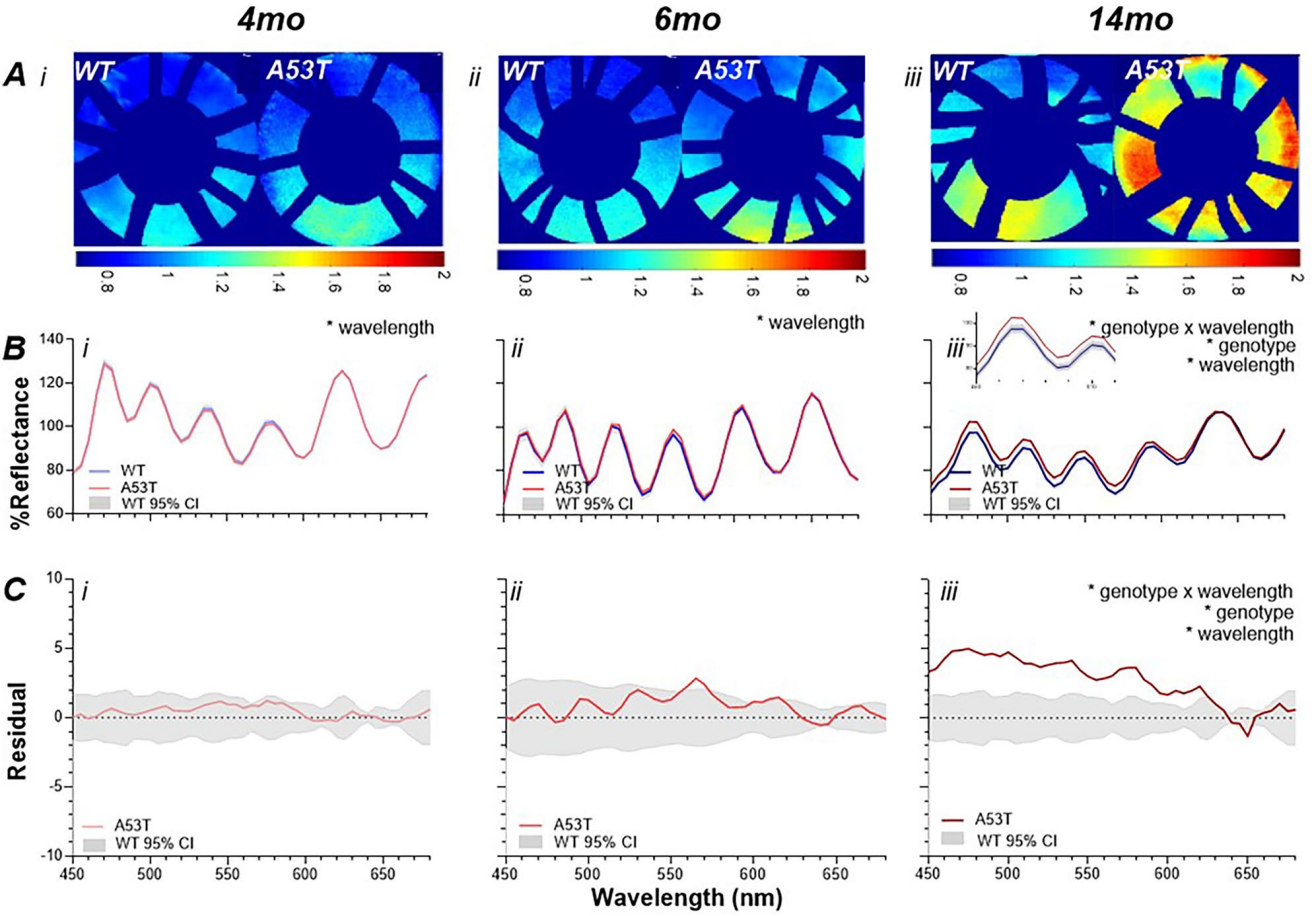
Hyperspectral imaging in A53T mice. (**A**) Representative retinal heat maps illustrate the difference ratio between short (450 to 480 nm) and long (630 to 660 nm) wavelengths. A53T mice exhibit warmer colours (higher ratio) compared to WT littermates particularly at the oldest age. For all panels WT and A53T comparisons are shown for the (i). 4-month-old (WT: light blue; A53T: light red) (ii). 6-month-old (WT: mid blue; A53T: mid red) (iii). 14-month-old cohort (WT: dark blue; A53T: dark red). (**B**) Average reflectance profiles of A53T (red) and WT (blue) mice with 95% CI (grey area). At (i) 4-month-old and (ii). 6-month-old a wavelength effect is found (*p* < 0.0001) and by (iii). 14-month-old an interaction effect is apparent (*p* < 0.0001), with a zoomed-in view (inset) of the reflectance at short wavelengths (460–520 nm) (**C**). Average residual values derived by subtracting the A53T group from the WT controls (red) with the variability in WT illustrated by grey 95% CI. No main effects are found at (i). 4-month-old nor (ii). 6-month-old but by (iii). 14-month-old a genotype x wavelength interaction (*p* < 0.0001) is apparent. Insert shows zoomed in changes at short wavelengths. *significance *p* < 0.05.

**Figure 3:**
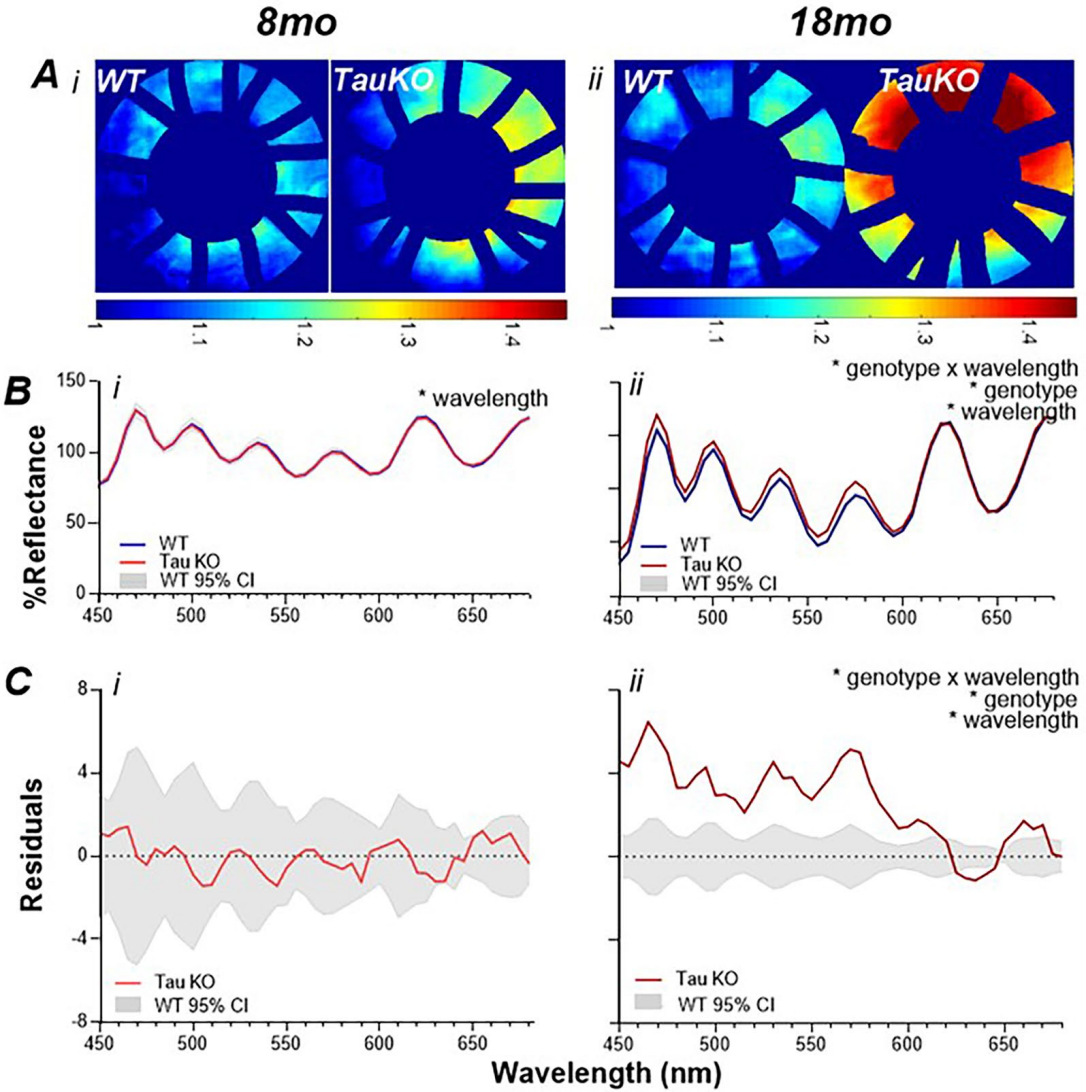
Hyperspectral imaging in TauKO mice. (**A**) Representative retinal heat maps illustrate the ratio between short and long wavelengths. TauKO mice exhibit warmer colours (higher ratio) compared to WT littermates at the oldest age. For all panels WT and TauKO comparisons are shown for the (i). 8-month-old (ii). 18-month-old cohort. (**B**) Average reflectance profiles of TauKO (red; 8-month-old: mid red; 18-month-old: dark red) and WT (blue; 8-month-old: mid blue; 18-month-old: dark blue) mice with 95% CI (grey area). At (i) 8-month-old a wavelength effect (*p* < 0.0001) is found and at (ii). 18-month-old an interaction effect (*p* < 0.0001) is observed, with a zoomed-in view (inset) of the reflectance at short wavelengths (460–520 nm) (**C**). By subtracting the TauKO group from the WT controls the residuals (red) indicate the same as Panel B. (i) In 8-month-old mice no interaction nor genotype effect is found and (ii) by 18-month an interaction effect is observed (*p* < 0.0001). Insert shows zoomed in changes at short wavelengths. 95% CI of WT in grey area, *significance *p* < 0.05.

“Residual” spectra were calculated by subtracting the average reflectance value of the normalized wild type (WT) spectrum at each wavelength, to quantify reflectance differences due to disease. A singular HSI metric was calculated as a ratio between short (450–480 nm) and long (630–660 nm) wavelengths to enable plotting of heatmaps (Matlab, version 9.6 (R2019a) The Mathworks, Inc., Natick, MA, USA) and multivariate correlation with other parameters. A 30 nm band was used to encompass an instrument pellicle-induced full oscillation in order to decrease bias induced by a singular wavelength. The 450–480 nm band was chosen given this is where the maximal PD and aging changes were observed (Figs. 2, 3, and 5) consistent with previous studies in AD^5,7,8^. The 630-660 nm band was chosen as an isobestic reference given previous studies find in in vitro^5,7^ and in vivo^7,8^ preparations of AD, less biological scatter occurs at these longer wavelengths (> ~ 600 nm).

### Retinal tissue analysis

Retinal levels of α-synuclein were quantified using western blot analysis. As A53T are genetically modified to express human native α-synuclein, toxic phosphorylated α-synuclein (pSer129 α-synuclein) and mouse α-synuclein were tested. The methodology and A53T data has been previously reported in Tran et al.^23^ (a subset *n* = 4–6 per age/phenotype included in this study) and the same approach was employed for TauKO tissue (*n* = 4–6 per age/phenotype). In brief, retinal tissue samples were homogenised in radioimmunoprecipitation assay (RIPA) lysis buffer, 50 µg electrophoresed on 4–12% polyacrylamide gels (NuPAGE™, Invitrogen, Thermo Fisher Scientific) and proteins transferred onto 0.45 μm PVDF membrane (Immobilon-P, Merck, Sigma-Aldrich). Prior to blocking the membrane in tris-buffered saline (TBS, Tris 20 mM, NaCl 150 mM, pH 7.6) containing 5% skim milk, Ponceau S staining solution (Thermo Fisher Scientific) was used to capture total protein and quantified using the ChemiDoc MP imaging system (BioRad, Hercules, California, USA). Membranes were incubated at room temperature with primary and secondary antibodies for 1 h each. All antibodies, human α-synuclein, 1:1000, #ab138501 [MJFR1], Abcam, Cambridge, UK; pSer129 α-synuclein 1:1000, #ab209422 [EP1536Y], Abcam; mouse α-synuclein, 1:2000, #D37A6, Dako, Glostrup, Denmark; tyrosine hydroxylase (TH), 1:1000, #AB152, Merck, Kenilworth, New Jersey, USA) were diluted in blocking buffer above. All washes were carried out using 0.01% Tween-20 (TBS-T). Enhanced chemiluminescence (ECL, Clarity Kit, BioRad) was used to detect protein bands which were visualized with the ChemiDoc MP imaging system (BioRad) and quantified by densitometry (ImageLab 6.1, BioRad). The relative protein abundance of each cell lysate was normalized to the respective lane’s automated TP measurement via ChemiDoc stain-free detection software.

Iron levels in the retina (including RPE) were quantified using inductively coupled plasma mass spectrometry approaches similar to those previously described in our group in brain tissue^33^. In a subset of animals (*n* = 3–8 per age/genotype group), retinal samples were weighed, lyophilized, and digested overnight in nitric acid (HNO_3_) (65% Suprapur, Merck). The samples were then heated at 90 °C for a 20 min period. Hydrogen peroxide (H_2_O_2_) (30% Aristar, BDH) of equal volume to the sample was added and left for 30 min, then heated for 15 min at 70 °C. Samples were then diluted with 1% HNO_3_. An Agilent 7700 series ICPMS instrument measured iron levels under routine multi-element operating conditions and a helium reaction gas cell. Standard calibration solutions used were ICP-MS-CAL2-1, ICP-MS-CAL-3 and ICP-MS-CAL-4, Accustandard. For internal control, a certified standard solution containing 200 ppb of Yttrium (Y89) was used (ICP-MS-IS-MIX1-1, Accustandard). Iron levels are expressed as microgram of metal per gram of dry tissue weight (µg/g).

Optical coherence tomography (OCT, Spectralis^®^, Heidelberg Engineering, Heidelberg, Germany) was used to quantify RNFL thickness in vivo as previously reported^23^. A subset of the same A53T RNFL data is analysed in the current study (*n* = 14–26 per group) and the same analysis conducted in Tau KO (*n* = 8–15 per group) and C57blk6J mice (*n* = 8–9 per group). In brief, retinal volume scans (8.1 × 8.1 × 1.9 mm) centred on the optic nerve head were acquired. OCT analysis. Automatic segmentation of the RNFL was conducted using the Heidelberg Eye Explorer 2 OCT reader plugin (Heyex, Heidelberg Engineering. The average RNFL thickness in the outer 6 mm Early Treatment Diabetic Retinopathy Study [ETDRS] ring was reported^23,34,35^.

### Statistical comparisons

Statistical tests were performed in Prism (version 8.0.2, Graphpad Software, Inc., La Jolla, CA, USA) and Matlab (version 9.6 (R2019a) The Mathworks, Inc., Natick, MA, USA). Repeated measures two-way analysis of variance (ANOVA) was applied, using Geisser-Greenhouse correction given that sphericity was not assumed with a repeated measures design^36^. Wavelength, age and genotype effects were examined, as well as interaction between these effects (genotype × wavelength; age × wavelength). Post hoc analysis was conducted using a Benjamini et al.^37,38^ approach which controls for the false discovery rate. Significance for *p* values was set at α = 0.05. All group data are presented as mean ± 95% confidence interval (CI).

Multivariate analysis was performed to determine how the HSI signal may be influenced by healthy aging, as well as key pathogenic PD factors that have shown altered reflectance using other spectral techniques and preparations, namely α-synuclein, iron and RNFL thickness. Multivariate analysis was carried out by fitting a linear model to predict the HSI ratio (450–480 nm/630–660 nm). The “fitlm” function from MATLAB R2019a was used to find a least squares solution. The multivariate predictors always included age, and one of either α-synuclein levels, iron levels, or RNFL thickness (i.e. a maximum of two predictors were included in any given model). We have not included results using more predictors combined, as sample sizes were reduced because not all of the animals had all assays done. For each model presented below, we frame the results in the context of the improvement in the fit quality, as assessed by the R^2^ value, for the two-predictor model compared with an appropriate univariate model (e.g. with just age as the predictor).

### Ethics approval and consent to participate

All experimental procedures involving animals were performed with approval from the Howard Florey Institute Animal Ethics Committee (Approval Numbers: 17-046-UM, 18-072-UM, 21-019-UM).

## Results

### Hyperspectral imaging in an α-synuclein overaccumulation mouse model

Figure 2 illustrates hyperspectral changes across the early (4-month-old), mid (6-month-old) and late (14-month-old) stages in the A53T and WT cohorts. The representative heat maps show that at 4- and 6-month-old the A53T retina appear similar to WT controls but that at 14-month-old the heatmaps exhibit considerably warmer colours in the A53T group. This agrees with the group spectrum data shown in Fig. 2B where the WT mice (blue line with grey shading representing the 95% CI) are compared with the age-matched A53T mice (red line) at 4-month-old (light blue vs. light red), 6-month-old (mid blue vs. mid red) and 14-month-old (dark blue vs. dark red). Here it can be seen that at 4-month-old the WT and A53T data overlap and no significant genotype changes are found (interaction *p* > 0.9999; genotype *p* = 0.7050; see Supplementary Table 1). A significant wavelength effect was found (*p* < 0.0001) which was expected given the inherent changes in retinal reflectance across the spectrum. The similarity in spectra between treated and control can be more clearly visualised in Fig. 2C where the data is expressed as “residuals”, with A53T—WT plotted. Here it can be seen that the average A53T residuals lie within the 95% CI of the WT controls and no genotype nor wavelength effects are found (interaction *p* > 0.9999; wavelength *p* > 0.9999; genotype *p* = 0.7050; see Supplementary Table 1). A similar story can be seen in the 6-month-old group with the WT and A53T groups largely overlapping in Figs. 2Bii,Cii and no significant genotype effects found (interaction *p* = 0.9832; genotype *p* = 0.4746, Supplementary Table 2). In contrast by the advanced PD stage (14-month-old) shown in Fig. 2Biii the A53T group shows greater reflectance at short wavelengths (interaction *p* < 0.0001; wavelength *p* < 0.0001; genotype effect *p* = 0.0125, Supplementary Table 3). This can be more easily visualized in the residual figure (Fig. 2Ciii) where the group average A53T group shows greater reflectance predominantly at shorter wavelengths (450–585 nm post-hoc *p* < 0.05, Supplementary Table 3).

### Hyperspectral imaging in an iron overaccumulation mouse model

The hyperspectral profiles in another PD model, TauKO mice which lack the tau protein and exhibit iron overload and progressive substantia nigra cell degeneration, is illustrated in Fig. 3. The representative heat maps in Fig. 3A show that at the early stage of the disease (8-month-old) TauKO retina appear similar to age-matched WT controls however by the advanced stage of the disease (18-month-old) TauKO retina have a considerably greater reflectance ratio exhibited as warm colours compared with WT counterparts. This is in agreement with the group average reflectance profiles (Fig. 3B) and (treated–control) residual graphs (Fig. 3C), where at 8-month-old TauKO mice a significant wavelength effect was found in the average reflectance profiles (*p* < 0.0001) but no interaction (*p* = 0.9881) nor genotype (*p* = 0.9643). By 18 months of age, as for the advanced A53T mice, the TauKO mice (Fig. 3Bii,Cii) exhibited greater reflectance at short wavelengths. This manifested as a significant interaction *(p* < 0.0001), wavelength *(p* < 0.0001) and genotype (*p* = 0.0147) with post-hoc changes significant in some wavelengths between 450–580 nm (Supplementary Table 5).

### Hyperspectral imaging in healthy aging across three wild-type strains

**Figure 4:**
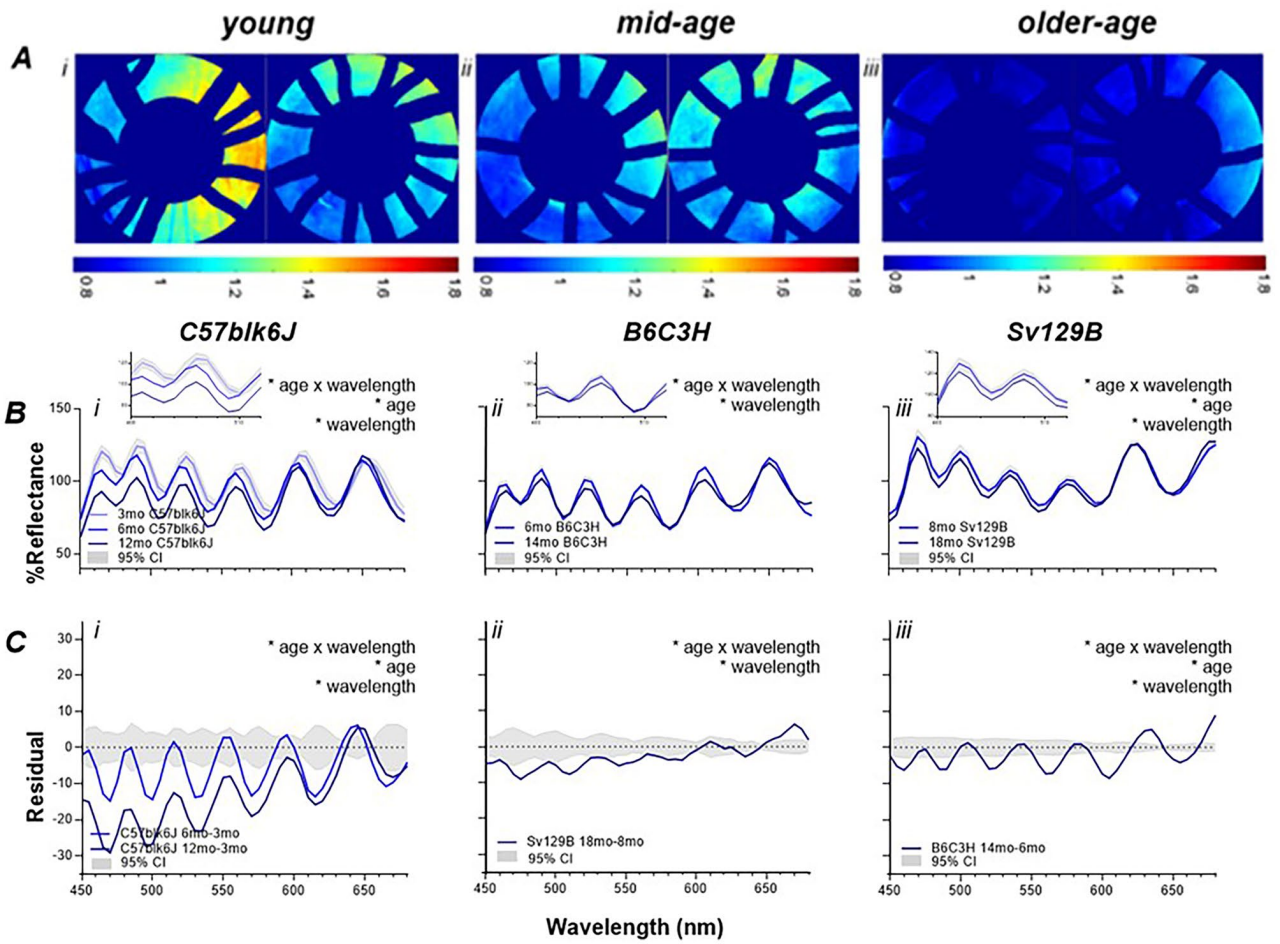
Hyperspectral imaging with healthy aging. (**A**) Representative retinal heat maps (ratio between short and long wavelengths) show that advancing age causes a lower ratio and hence cooler colours. In Panels (**B–E**), the following “control” cohorts are examined (**i**). C57blk6J mice at young (3-month-old; light blue), mid-age (6-month-old; medium blue) and older-age (12-month-old; dark blue) (**ii**). WT littermates of A53T mice (B6C3H background strain) at mid- (6-month-old; medium blue) and older-age (14-month-old; dark blue) (**iii**). WT littermates of TauKO mice (Sv129B background strain) at mid-age (8-month-old; medium blue) and older-age (18-month-old; dark blue). (**B**) Average reflectance profiles show that in all three control cohorts advancing age causes an interaction effect (*p* < 0.0001) due to decreasing reflectivity at short wavelengths and increasing reflectivity at long wavelengths, with a zoomed-in view (insets) of the reflectance at short wavelengths (460–520 nm) (**C**). Similarly, the residual figures (older age—younger age) illustrate the same effect (interaction, *p* < 0.0001). Insert shows zoomed in changes at short wavelengths. *significance *p* < 0.05.

In order to compare the effects of normal healthy age-related changes on HSI against a PD phenotypes, control animals were examined. Figure 4 illustrates 3 separate cohorts of control mice. The left column (Fig. 4Bi,Ci) illustrates the changes with healthy aging in C57blk6J mice (WT littermates of 5xFAD mice) and show that with advancing age there is reduced reflectance at short wavelengths (Fig. 4Bi, interaction *p* < 0.0001). This is the opposite pattern to that observed in the PD models (A53T Fig. 2; TauKO Fig. 3). This manifested as a significant interaction effect in the average reflectance profile (Fig. 4Bi, *p* < 0.0001) and treated—control residual graphs (Fig. 4Ci,* p* < 0.0001) with post hoc significance differences between groups at shorter wavelengths largely below 625 nm (Supplementary Table 6).

Similarly, to further examine the effects of healthy aging, the WT littermates of A53T mice at different ages (6-month-old and 14-month-old) were compared (middle column) as were the WT littermates of TauKO mice at different ages (8-month-old and 18-month-old) (right column). A similar effect was observed in both WT groups to that found in C57blk6J mice with advancing age. More specifically, a decreased reflectance at short wavelengths was noted (WT of A53T: Fig. 4Bii, interaction *p* < 0.0001; Fig. 4Cii* p* < 0.0001, WT of Tau KO: Fig. 4Biii, interaction *p* < 0.0001; Fig. 4Ciii p < 0.0001. Post hoc analyses in Supplementary Tables 7, 8).

### Multivariate correlations of hyperspectral imaging to examine mechanism

**Figure 5:**
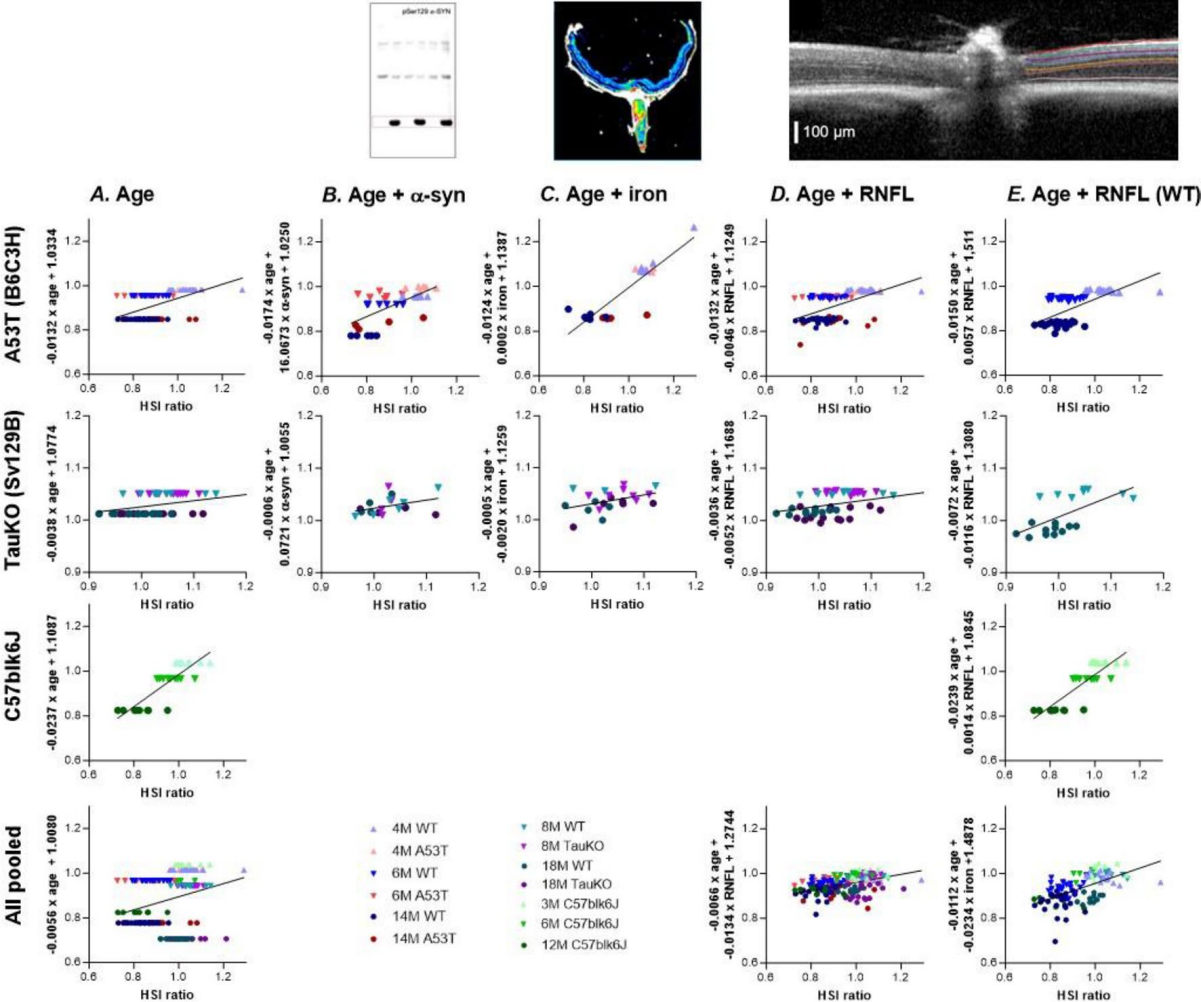
Multivariate correlations between HSI and age, α-synuclein, iron and RNFL. Representative images of co-factors and their method of analysis show in top row. α-synuclein assessed with western blot, from Tran et al.^23^; iron assessed with mass spectrometry; RNFL quantified via OCT, from Tran et al.^23^ (**A**). HSI ratio correlated against age (**B**). HSI ratio correlated against age and α-synuclein. In A53T mice toxic phosphorylated α-synuclein is plotted and in TauKO animals, mouse α-synuclein is plotted (**C**). HSI ratio correlated against age and iron (**D**). HSI ratio correlated against age and RNFL (**B**). HSI ratio correlated against age and RNFL in control animals only (healthy aging).

To investigate whether α-synuclein, iron or RNFL thinning were contributing to the hyperspectral changes found in the PD animal models these were quantified in retinal tissue. As healthy aging also caused a change in hyperspectral profile, multivariate analyses were conducted comparing HSI ratio (450–480 nm/630–660 nm) against aging and examined whether this relationship improved with the addition of α-synuclein, iron or RNFL as a co-factor. This improvement is indicated by an improvement in the R^2^ value (warmer to cooler colours) in the continued presence of a significant *p *value. Table 1 and Fig. 5 show that there is a significant relationship between HSI ratio and age (Table 1A, Fig. 5A) across all groups individually and when pooled (*p* < 0.05). When α-synuclein is added as a cofactor (Table 1B, Fig. 5B) this improves the correlation in A53T mice than just age alone (R^2^ = 0.3141) and the relationship with toxic α-synuclein is marginally more (R^2^ = 0.4302) than the combination of all α-synuclein in the retina (R^2^ = 0.4099). Similarly, with the addition of iron (Table 1C, Fig. 5C) as a co-factor the correlation in A53T mice is improved (R^2^ = 0.4251) over age alone. In the TauKO mice when α-synuclein or iron were added as a co-factor the relationship was not significant (*p* = 0.0551 to 0.0945). When RNFL is added as a cofactor to age (Table 1D, Fig. 5D) the multivariate correlation improves when all animals are pooled together, and even more so when only WT mice are analysed (Table 1E, Fig. 5E) as evidenced by an improvement in R^2^ in most of groups examined (cooler highlight colours).

**Table 1:**
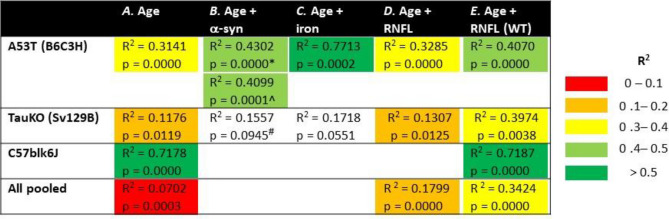
Multivariate correlations between HSI and age, α-synuclein, iron and RNFL.

Significant relationships (p < 0.05) are highlighted in different colours according to the R^2^ value with R^2^ = 0 to 0.1 (red), R^2^ = 0.1 to 0.2 (orange), R^2^ = 0.3 to 0.4 (yellow), R^2^ = 0.4 to 0.5 (light green), R^2^ =  > 0.5 (dark green). A53T mice underwent multivariate correlation against toxic phosphorylated α-synuclein (*) and total α-synuclein (^, human native α-synuclein + toxic α-synuclein + mouse α-synuclein). TauKO mice only possess endogenous rodent α-synuclein and thus were correlated against retinal mouse α-synuclein levels (^#^).

### Comparison of hyperspectral imaging across Parkinson’s, Alzheimer’s and healthy aging mouse models

**Figure 6:**
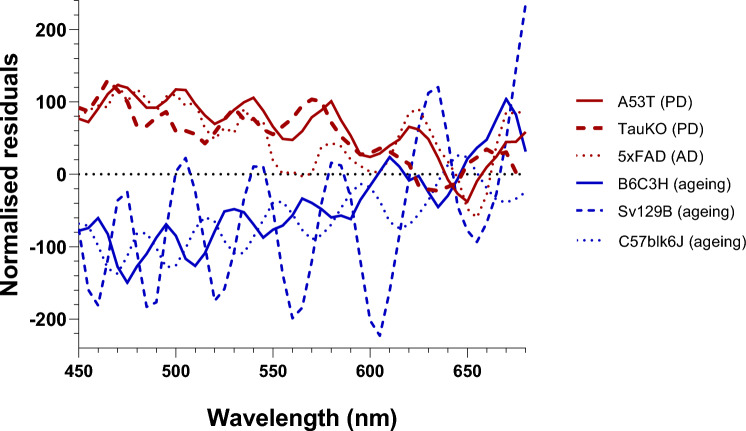
Normalized residual plots highlighting the PD/AD versus aging phenotypes. Both A53T and TauKO PD models show a similar hyperspectral pattern to the 5xFAD AD model, with increased reflectance below ~ 600 nm. The hyperspectral effect in the aged control mice is the mirror opposite to this with decreased reflectance shown below ~ 600 nm across 3 different mouse strains.

To compare the shape of the different conditions examined, the residual plots were normalized to a short wavelength band where maximal PD and aging changes were observed (450–480 nm; Fig. 6). This illustrates that in the two PD models examined at the older age (A53T 14-month-old; TauKO 18-month-old) that the hyperspectral profile is similar in these conditions. Moreover, the pattern is also comparable to an AD model, the 5xFAD mouse (12-month-old). In contrast healthy aging shows the mirror opposite trend in three strains of mice, B6C3H (A53T WT littermates, 14–6-month-old), Sv129B (WT littermates of TauKO, 18–8-month-old) and C57blk6 (WT littermates of 5xFAD, 12–3-month-old).

## Discussion

In this study we demonstrate that two mouse models of PD exhibit similar hyperspectral profiles that are opposite to those observed with healthy aging. The A53T and TauKO mouse models show a hyperspectral effect at later stages of the disease that manifests as increased reflectivity at shorter wavelengths in the visible spectrum. In contrast, healthy aging alone in three cohorts of wild-type mice show the opposite effect to Parkinson’s related changes, namely a decreased reflectivity at short wavelengths which amplifies with advancing age. Multivariate analyses indicate that in addition to age, retinal levels of α-synuclein and iron may contribute to HSI metrics as does thickness of the RNFL layer. In combination, this data illustrates for the first time a PD hyperspectral profile distinct from healthy aging. This presents as an exciting non-invasive imaging modality to examine PD, given that brain imaging tools are not yet commercially available to assay α-synuclein nor iron and cerebrospinal fluid requires invasive lumbar puncture.

### Hyperspectral imaging in Parkinson’s disease

Our HSI data suggests that in the A53T mouse model there is a wavelength dependent Parkinson's effect on reflectance. This difference presents in the late (14-month-old) disease stage as a linear decreasing trend with wavelength, resulting in increased reflectance at lower wavelengths (shorter than 585 nm) of incident light compared to WT littermates (Fig. 2Ciii, Supplementary Table 3). In comparison the 4-month-old and 6-month-old A53T data depicts an A53T reflectance profile which lies within the 95%CI of the WT littermates, suggesting that this wavelength dependent change in the A53T model presents only at advanced disease stages (Fig. 2; Supplementary Tables 1–3). These age-related changes are in accordance with the literature and our work^23^ where western blot assessment of α-synuclein in the retina (pSer129) plateaus between 8 and 18 months of age but is similar to control at 4 to 6 months of age. This is also in agreement with studies from our group and others showing that the motor phenotype and cortical α synuclein changes plateau between 8 and 16 months of age.

Similar hyperspectral effects were found in the TauKO model with an increased reflectance at short wavelengths, below 580 nm (Fig. 3, Supplementary Table 5). Again, the difference presented in the later disease stage (18-month-old) paralleling when motor dysfunction manifests (between 12 to 24 months^27^), α-synuclein levels increase in the caudate putamen (15-month-old^28^) and Nissl stereological counts and tyrosine hydroxylase stained neurons decrease in the substantia nigra (12 months^27^). At earlier prodromal stages of the disease a hyperspectral effect is not observed (8-month-old) which again parallels the overall disease stage with a lack of motor impairment (i.e. 6 to 7-month-old^27,28^) and cortical α synuclein noted at similar ages. It is worth noting that the reflectance heat maps show non-uniform reflectance across the retina. It is likely that the spatial heterogeneity may be related to the spatial distribution of the driving factor, e,g., spatial distribution of a-synuclein/iron or RNFL thinning, Further study to co-localize these heat maps with flat mounted tissue stained with α-synuclein or spatial mapping of RNFL thinning is warranted to understand the non-uniform reflectance across the retina. An additional improvement to the processing of hyperspectral images in future work would be to normalize the intensity recorded at each wavelength according to the spectral output of the source. This was not undertaken here, as the significant non-uniformity imposed on the spectrum by the pellicle beam-splitter complicates this task (especially as it is sensitive to small rotations of the beam-splitter). However, as the major aim is to compare the wild-type to the disease conditions, normalization of the spectra does not have any bearing on the conclusions of the study, which rely on making statistical comparisons between the two groups which each underwent identical processing steps.

Although the two Parkinson’s models showed similar hyperspectral profiles, healthy aging alone exhibited a distinct and opposing signature. As such given the complex interplay between these factors, to investigate the influence of PD driven pathobiological changes on HSI a multivariate analysis was conducted adding on α-synuclein or iron as a co-factor to age (Table 1). This demonstrated that in A53T mice, addition of α-synuclein improved the capacity of the model to explain the HSI ratio. This was the case for both the toxic phosphorylated form of α-synuclein and an overall measure of α-synuclein (summation of pSer129, human α-synuclein, mouse α-synuclein). Iron levels in the retina and RPE also improved the models’ predictive capacity for HSI ratio in A53T mice. Although the TauKO mice did not show marked improvements with the addition of α-synuclein or iron as a co-factor for multivariate analyses, these multivariate data sets had lower sample size (A53T *n* = 28 to 33; TauKO *n* = 19 to 22) and a decreased spread of data (no “young” age group represented by a narrower axis in Fig. 5) than the A53T counterparts. Thus, future investigation in larger sample sizes at a broader range of ages is warranted in TauKO mice.

This suggestion that retinal α-synuclein and iron might be the underlying biology that drive Parkinson’s related HSI is supported by the literature which has examined other forms of retinal spectral imaging in in vitro or ex vivo preparations. Oh et al.^13^ used a human neuroblastoma cell line which was treated with 1-methyl-4-phenylpyridinium (MPP+) and ferric ammonium citrate that caused iron accumulation. They imaged these cell culture preparations with a dark-field HSI system that spanned 400 to 1000 nm wavelengths. They found that imaging isolated iron within a water mixture (ferric ammonium citrate) exhibited absorption in a bell-shaped curve. These iron depositions within their cell culture model showed absorption largely occurred between ~ 475 and 620 nm (EC_50_ estimates) with a peak at ~ 535 nm. In contrast, the glass plate showed no reflectance and the cellular areas without iron were much less reflectile. These findings are in agreement with the current study which shows increased reflectance across a similar wavelength band (~ 450–600 nm). The opposing polarity is observed between studies because Oh et al.^13^ used dark field microscopy (thus manifesting as increased absorbance) and the current study employed an ophthalmoscopic system more akin to bright field microscopy (manifesting as increased reflectance). This sign inversion from absorption to reflectance has previously been shown in hyperspectral studies of Alzheimer’s retinae.^7^

The presence of proteins in the retina have been implicated to increase light scatter in the visible spectrum range. This has been shown for amyloid-beta (implicated in AD) in cell culture^5^, within ex vivo^5^ and in vivo mouse preparations^7,8^ as well as human patients^6^.This has been shown for amyloid-beta (implicated in AD) in cell culture^5^, within ex vivo^5^ and in vivo mouse preparations^7,8^ as well as human patients^6^. α-synuclein (implicated in PD) is another protein which has shown increased light scattering properties. In vitro preparations of native α-synuclein exhibit Rayleigh scatter when an excitation wavelength of 350 nm was used and an emission band recorded across 300 to 400 nm Rayleigh scattering occurs when light interacts with scatterers smaller than ~ 1/10th the wavelength of incident light and may explain the increased reflectance at shorter wavelengths within the current hyperspectral study. Mammadova et al.^10^ used a different spectroscopy approach (Raman spectroscopy) on retinal cross sections from A53T mice. Raman spectroscopy uses a single monochromatic wavelength (532 nm) and measures scatter when it passes through a substance at additional frequencies that are distinct from the excitation wavelength. This approach also differentiated between age-matched control and A53T mice within ex vivo samples. These studies differ in their spectrometry approach to the current study (hyperspectral imaging) but further highlight the light scattering properties of α-synuclein.

The current study shows an in vivo hyperspectral profile from PD retinae that are distinct from control. Collectively, previous literature and the current study indicate that the presence of α-synuclein and iron in the retina may influence HSI. Future interventional studies which pharmacologically decrease tissue levels of α-synuclein or iron would further aide our understanding of the factors which drive HSI.

### Hyperspectral imaging with healthy aging

Three separate cohorts of wild-type mice indicate that with advancing age there is less reflectivity at shorter wavelengths, largely below 600 nm, with increasing spectral differences the shorter the wavelength (Fig. 4, Supplementary Tables 6–8). The magnitude of this reduced reflectance was more pronounced the greater the difference in age between the groups compared.

Aging in the eye can present in several different ways including vitreous liquefaction, cataracts, inflammation, oxidative stress and lipofuscin accumulation^39^. In particular, several aging changes in the eye can reduce short wavelength reflectance. Age-related lenticular changes cause a reduction of light transmission, particularly in the shorter wavelength bands (~ 400–500 nm) as the yellowing of the lens promotes blue light absorption^39–41^. Within HSI this would manifest as a decreased hyperspectral reflectance with worsening lens changes, particularly at shorter wavelengths.

One way to assess the effect of age-related lens changes on HSI may be to compare phakic and pseudophakic patients. However as commonly employed intraocular lenses (IOLs) have yellow-tinting, blue light and UV blocking properties this may account for a similar hyperspectral profile in 10 patients who were examined before and after cataract surgery^6.^ This is advantageous from a clinical implementation point of view as it would indicate that HSI may be used in patients with or without their cataracts removed. To directly answer whether the aging lens may be contributing to the decreased reflectance seen in this study would require removal of the rodent lens and replacement with a transparent IOL that does not possess any yellow tinting/blue light filters. Comparison of HSI before and after cataract surgery in human patients with transparent IOLs used may also elucidate the clinical utility here.

Previous studies in animal models and humans have shown that the RNFL is reflectile at short wavelengths^14–16,42,43^. Addition of RNFL as a cofactor to age with both WT and PD groups did not markedly improve multivariate correlations in individual strains of mice, but an improvement in R^2^ was observed when all groups were pooled together (Table 1A, D). In contrast, when only WT animals were analyzed, thereby examining the effect of RNFL influencing healthy aging alone, RNFL as a cofactor improved most of the correlations (Table 1A, E). One explanation for this is that when PD and control groups are combined there are multiple factors at play (including PD driven α-synuclein/iron deposition as well as age-induced RNFL thinning) whereas with advancing age alone, RNFL alterations play a more dominant role in influencing hyperspectral outcomes. To examine this a multivariate analysis which takes into account all factors would be ideal. Although not possible in the current study due to limited sample size (HSI, α-synuclein, iron, RNFL were only examined in *n* = 5 to 8 overlapping animals) this remains an area for future investigation.

If RNFL thinning and/or lenticular changes are driving the aging changes seen (Fig. 4), given that these are commonly assessed at eyecare clinics, in the future correcting for these masking effects would further increase sensitivity of HSI to differentiate PD from control subjects.

### Comparison of Parkinson’s disease hyperspectral imaging with Alzheimer’s disease

Similar HSI reflectance changes were found in an AD (5xFAD) mouse model and the two PD mouse models (A53T, TauKO) examined in this study. All groups exhibit an increase in reflectance at short wavelengths (Fig. 6). Figure 5 suggests that levels of α-synuclein and iron may be playing a role in the increased reflectance found in the PD models at short wavelengths. It may also be possible that changes in the collagen network alters reflectance at this wavelength band^44^. Although it has been shown that collagen hydrogels improve brain repair in a rat model of PD^45^, how the collagen network alters in these PD mouse models and whether it affects HSI reflectance require further investigation.

As discussed above, the trends in PD mouse models were opposite to those produced by normal aging (Fig. 6). The similar generalized trends between AD and PD are consistent with the idea that small proteinopathies in both conditions may be producing an increase in light scattering and thus reflectance at low wavelengths. At present, HSI in AD is better characterized than in PD^5–8^. Indeed, the AD mouse model employed here (5xFAD) has been shown to exhibit similar HSI metrics to mild cognitive impairment patients who have high brain amyloid-beta load compared with age-matched control counterparts as well as to in vitro preparations of amyloid beta^7^. As such it is possible that that the HSI signature observed may be a general proteinopathy biomarker, given the overlap between AD and PD. Clinically, AD and PD are easily differentiated (predominantly cognitive versus motor symptoms) and thus this overlap does not blunt the future utility of HSI as an indicator for PD.

## Conclusions

The current study is the first to examine HSI in a live mouse Parkinsonian retina. Hyperspectral imaging in two mouse PD models show a signature which is similar to an AD mouse model but completely distinct and opposite to that arising from healthy aging. We show preliminary evidence for an association of HSI with retinal levels of α-synuclein and iron. Furthermore, in aging, the integrity of the RNFL may influence HSI. Future studies which more comprehensively examine the biological drivers of PD and age-induced hyperspectral changes will pave the way for HSI as a non-invasive, scalable biomarker for neurodegenerative disease.

### Supplementary Information

Supplementary Information.

## Data Availability

The authors confirm that we will adhere to the journals data availability policies including making materials, data and associated protocols promptly available to readers without undue qualifications in material transfer agreements. Please contact corresponding author at christine.nguyen@unimelb.edu.au.

## Abbreviations

5xFAD: 5 familial human AD genes
A53T: HA53T transgenic line M83
AD: Alzheimer’s disease
ANOVA: Analysis of variance
α-synuclein: Alpha-synuclein
CI: Confidence intervals
ETDRS: Early treatment diabetic retinopathy study
HSI: Hyperspectral imaging
MPP+: 1-Methyl-4-phenylpyridinium
PD: Parkinson’s disease
RPE: Retinal pigment epithelium
RNFL: Retinal nerve fibre layer
ROI: Region of interest
OCT: Optical coherence tomography
RIPA: Radioimmunoprecipitation assay (RIPA) lysis buffer
sCMOS: Scientific complementary metal oxide semiconductor
TauKO: Tau knock out
TH: Tyrosine hydroxylase
WT: Wild type
