# Supplementary material for: Retinal hyperspectral imaging in mouse models of Parkinson’s disease and healthy aging
Source: Sci Rep. 2024 Jul 12;14:16089. doi: 10.1038/s41598-024-66284-7 (PMC11245556; doi:10.1038/s41598-024-66284-7)
Supplement: Supplementary file 1 — Supplementary Information. [file 41598_2024_66284_MOESM1_ESM.docx]

Supplementary Material

**Retinal hyperspectral imaging in mouse models of Parkinson’s disease and healthy aging**

*Paul Trlin^1^, Jenny Gong^1^, Katie K.N. Tran^1^, Vickie H.Y. Wong^1^, Pei Ying Lee^1^, Anh Hoang^1^, Da Zhao^1^, Leah C. Beauchamp^2,3^, Jeremiah K. H. Lim^1,4^, Andrew Metha^1^, Kevin J. Barnham^2^, David I. Finkelstein^2^, Bang V. Bui^1^, Phillip Bedggood^1^, Christine T.O. Nguyen^1*^*

^1^ Department of Optometry and Vision Sciences, University of Melbourne, Parkville, 3010, Victoria, Australia.

^2^Florey Institute of Neuroscience and Mental Health, Parkville, 3010, Victoria, Australia.

^3^Ann Romney Center for Neurologic Diseases, Brigham and Women's Hospital & Harvard Medical School, Boston, 02115, Massachusetts, USA (current affiliation).

^4^Discipline of Optometry, School of Allied Health, University of Western Australia, Crawley, 6009, Western Australia, Australia (current affiliation).

*** Correspondence:** Christine T.O. Nguyen: [christine.nguyen@unimelb.edu.au](mailto:christine.nguyen@unimelb.edu.au)

## Supplementary Figures


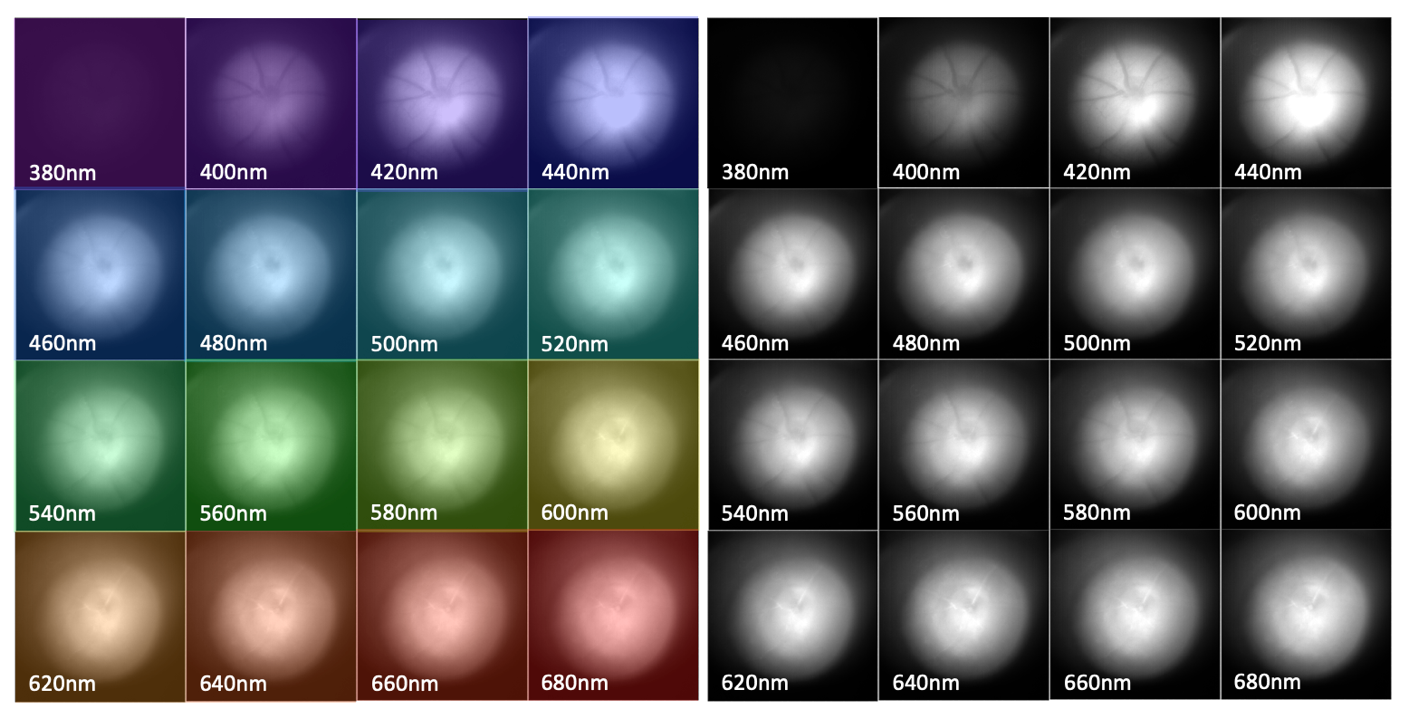


**Supplementary Figure 1.** **Representative retinal images at different wavelengths.** Hyperspectral images were obtained by illuminating varying wavelengths of incident light onto the retina. A representative retina is illustrated across the spectrum, with 20 nm increments shown. Images pseudo-coloured to visually represent the imaging wavelength used. Of note certain particular wavelengths highlight certain features in the retina (*i.e.* blood vessels easily visible around 560 nm).

**Supplementary Table 1. Statistics for 4-month-old WT vs A53T mice**

Two-way repeated measures ANOVA where, DF (degrees of freedom), SS (sum of squares), MS (mean square), and post-hoc comparisons between 4-month-old WT and A53T mice **(A)** % reflectance, and **(B)** residual

| 1. **% reflectance** | SS | DF | MS | F (DFn, DFd) | P-value |
| --- | --- | --- | --- | --- | --- |
| Wavelength x Genotype | 65.71 | 46 | 1.429 | F (46, 1288) = 0.1470 | P>0.9999 |
| Wavelength | 246709 | 46 | 5363 | F (46, 1288) = 551.7 | P<0.0001 |
| Genotype | 57.47 | 1 | 57.47 | F (1, 28) = 0.1462 | P=0.7050 |
| Subject | 11003 | 28 | 393.0 | F (28, 1288) = 40.43 | P<0.0001 |
| Residual | 12520 | 1288 | 9.720 |  |  |

| Two-stage linear step-up procedure of Benjamini, Krieger and Yekutieli | Mean Diff. | Discovery | P-Value |
| --- | --- | --- | --- |
| 450 | -0.007550 | No | 0.9961 |
| 455 | 0.2526 | No | 0.8701 |
| 460 | -0.06518 | No | 0.9663 |
| 465 | 0.07401 | No | 0.9618 |
| 470 | 0.4339 | No | 0.7787 |
| 475 | 0.6470 | No | 0.6752 |
| 480 | 0.4448 | No | 0.7733 |
| 485 | 0.2527 | No | 0.8700 |
| 490 | 0.2149 | No | 0.8893 |
| 495 | 0.3558 | No | 0.8177 |
| 500 | 0.5269 | No | 0.7330 |
| 505 | 0.6938 | No | 0.6532 |
| 510 | 0.8469 | No | 0.5834 |
| 515 | 0.5102 | No | 0.7411 |
| 520 | 0.4828 | No | 0.7545 |
| 525 | 0.4596 | No | 0.7660 |
| 530 | 0.6913 | No | 0.6544 |
| 535 | 0.9103 | No | 0.5555 |
| 540 | 1.053 | No | 0.4955 |
| 545 | 1.175 | No | 0.4469 |
| 550 | 0.9523 | No | 0.5375 |
| 555 | 0.9551 | No | 0.5362 |
| 560 | 0.6826 | No | 0.6585 |
| 565 | 0.8750 | No | 0.5709 |
| 570 | 0.8336 | No | 0.5893 |
| 575 | 1.224 | No | 0.4280 |
| 580 | 1.027 | No | 0.5061 |
| 585 | 1.058 | No | 0.4935 |
| 590 | 0.7494 | No | 0.6274 |
| 595 | 0.4586 | No | 0.7665 |
| 600 | 0.04285 | No | 0.9779 |
| 605 | -0.2385 | No | 0.8772 |
| 610 | -0.2201 | No | 0.8866 |
| 615 | -0.1546 | No | 0.9203 |
| 620 | -0.2566 | No | 0.8680 |
| 625 | 0.1182 | No | 0.9390 |
| 630 | 0.4653 | No | 0.7631 |
| 635 | 0.1429 | No | 0.9263 |
| 640 | 0.1442 | No | 0.9256 |
| 645 | 0.05027 | No | 0.9740 |
| 650 | -0.2259 | No | 0.8837 |
| 655 | -0.2833 | No | 0.8544 |
| 660 | -0.2934 | No | 0.8493 |
| 665 | -0.01218 | No | 0.9937 |
| 670 | 0.02968 | No | 0.9847 |
| 675 | 0.2926 | No | 0.8497 |
| 680 | 0.6096 | No | 0.6930 |

| 1. **residual** | SS | DF | MS | F (DFn, DFd) | P-value |
| --- | --- | --- | --- | --- | --- |
| Wavelength x Genotype | 65.71 | 46 | 1.429 | F (46, 1288) = 0.1470 | P>0.9999 |
| Wavelength | 65.71 | 46 | 1.429 | F (46, 1288) = 0.1470 | P>0.9999 |
| Genotype | 57.47 | 1 | 57.47 | F (1, 28) = 0.1462 | P=0.7050 |
| Subject | 11003 | 28 | 393.0 | F (28, 1288) = 40.43 | P<0.0001 |
| Residual | 12520 | 1288 | 9.720 |  |  |

| Two-stage linear step-up procedure of Benjamini, Krieger and Yekutieli | Mean Diff. | Discovery | P-Value |
| --- | --- | --- | --- |
| 450 | 0.007550 | No | 0.9961 |
| 455 | -0.2526 | No | 0.8701 |
| 460 | 0.06518 | No | 0.9663 |
| 465 | -0.07401 | No | 0.9618 |
| 470 | -0.4339 | No | 0.7787 |
| 475 | -0.6470 | No | 0.6752 |
| 480 | -0.4449 | No | 0.7733 |
| 485 | -0.2527 | No | 0.8700 |
| 490 | -0.2149 | No | 0.8893 |
| 495 | -0.3558 | No | 0.8177 |
| 500 | -0.5269 | No | 0.7330 |
| 505 | -0.6938 | No | 0.6532 |
| 510 | -0.8469 | No | 0.5834 |
| 515 | -0.5102 | No | 0.7411 |
| 520 | -0.4828 | No | 0.7545 |
| 525 | -0.4596 | No | 0.7660 |
| 530 | -0.6913 | No | 0.6544 |
| 535 | -0.9103 | No | 0.5555 |
| 540 | -1.053 | No | 0.4955 |
| 545 | -1.175 | No | 0.4469 |
| 550 | -0.9523 | No | 0.5375 |
| 555 | -0.9551 | No | 0.5362 |
| 560 | -0.6826 | No | 0.6585 |
| 565 | -0.8750 | No | 0.5709 |
| 570 | -0.8336 | No | 0.5893 |
| 575 | -1.224 | No | 0.4280 |
| 580 | -1.027 | No | 0.5061 |
| 585 | -1.058 | No | 0.4935 |
| 590 | -0.7494 | No | 0.6274 |
| 595 | -0.4586 | No | 0.7665 |
| 600 | -0.04285 | No | 0.9779 |
| 605 | 0.2385 | No | 0.8772 |
| 610 | 0.2201 | No | 0.8866 |
| 615 | 0.1546 | No | 0.9203 |
| 620 | 0.2566 | No | 0.8680 |
| 625 | -0.1182 | No | 0.9390 |
| 630 | -0.4653 | No | 0.7631 |
| 635 | -0.1429 | No | 0.9263 |
| 640 | -0.1442 | No | 0.9256 |
| 645 | -0.05027 | No | 0.9740 |
| 650 | 0.2259 | No | 0.8837 |
| 655 | 0.2833 | No | 0.8544 |
| 660 | 0.2934 | No | 0.8493 |
| 665 | 0.01219 | No | 0.9937 |
| 670 | -0.02968 | No | 0.9847 |
| 675 | -0.2926 | No | 0.8497 |
| 680 | -0.6096 | No | 0.6930 |

**Supplementary Table 2. Statistics for 6-month-old WT vs A53T mice**

Two-way repeated measures ANOVA where, DF (degrees of freedom), SS (sum of squares), MS (mean square), and post-hoc comparisons between 6-month-old WT and A53T mice **(A)** % reflectance, and **(B)** residual

| 1. **% reflectance** | SS | DF | MS | F (DFn, DFd) | P-value |
| --- | --- | --- | --- | --- | --- |
| Wavelength x Genotype | 219.5 | 46 | 4.771 | F (46, 1334) = 0.6054 | P=0.9832 |
| Wavelength | 231107 | 46 | 5024 | F (46, 1334) = 637.5 | P<0.0001 |
| Genotype | 241.5 | 1 | 241.5 | F (1, 29) = 0.5248 | P=0.4746 |
| Subject | 13344 | 29 | 460.2 | F (29, 1334) = 58.38 | P<0.0001 |
| Residual | 10514 | 1334 | 7.881 |  |  |

| Two-stage linear step-up procedure of Benjamini, Krieger and Yekutieli | Predicted (LS) mean diff. | Discovery | P-Value |
| --- | --- | --- | --- |
| 450 | -0.05713 | No | 0.9698 |
| 455 | 0.2006 | No | 0.8943 |
| 460 | -0.3632 | No | 0.8099 |
| 465 | -0.8107 | No | 0.5914 |
| 470 | -0.9885 | No | 0.5128 |
| 475 | -0.2639 | No | 0.8613 |
| 480 | 0.3462 | No | 0.8187 |
| 485 | 0.2037 | No | 0.8927 |
| 490 | -0.5053 | No | 0.7379 |
| 495 | -1.361 | No | 0.3676 |
| 500 | -1.272 | No | 0.3999 |
| 505 | -0.8240 | No | 0.5854 |
| 510 | -0.3497 | No | 0.8169 |
| 515 | -0.2229 | No | 0.8826 |
| 520 | -0.7779 | No | 0.6065 |
| 525 | -1.673 | No | 0.2682 |
| 530 | -2.004 | No | 0.1847 |
| 535 | -1.698 | No | 0.2611 |
| 540 | -1.347 | No | 0.3726 |
| 545 | -1.155 | No | 0.4445 |
| 550 | -1.300 | No | 0.3894 |
| 555 | -1.799 | No | 0.2337 |
| 560 | -2.282 | No | 0.1309 |
| 565 | -2.825 | No | 0.0616 |
| 570 | -2.416 | No | 0.1098 |
| 575 | -1.653 | No | 0.2739 |
| 580 | -1.178 | No | 0.4353 |
| 585 | -0.7378 | No | 0.6252 |
| 590 | -0.7482 | No | 0.6203 |
| 595 | -0.7821 | No | 0.6046 |
| 600 | -1.171 | No | 0.4380 |
| 605 | -1.114 | No | 0.4606 |
| 610 | -1.346 | No | 0.3730 |
| 615 | -1.460 | No | 0.3338 |
| 620 | -1.010 | No | 0.5037 |
| 625 | -0.4104 | No | 0.7858 |
| 630 | 0.08889 | No | 0.9531 |
| 635 | 0.3629 | No | 0.8101 |
| 640 | 0.5322 | No | 0.7246 |
| 645 | 0.4710 | No | 0.7551 |
| 650 | -0.2156 | No | 0.8865 |
| 655 | -0.4516 | No | 0.7649 |
| 660 | -0.7879 | No | 0.6019 |
| 665 | -0.8552 | No | 0.5712 |
| 670 | -0.3988 | No | 0.7917 |
| 675 | -0.1604 | No | 0.9154 |
| 680 | 0.1190 | No | 0.9372 |

| 1. **residual** | SS | DF | MS | F (DFn, DFd) | P-value |
| --- | --- | --- | --- | --- | --- |
| Wavelength x Genotype | 219.5 | 46 | 4.771 | F (46, 1334) = 0.6054 | P=0.9832 |
| Wavelength | 219.5 | 46 | 4.771 | F (46, 1334) = 0.6054 | P=0.9832 |
| Genotype | 241.5 | 1 | 241.5 | F (1, 29) = 0.5248 | P=0.4746 |
| Subject | 13344 | 29 | 460.2 | F (29, 1334) = 58.38 | P<0.0001 |
| Residual | 10514 | 1334 | 7.881 |  |  |

| Two-stage linear step-up procedure of Benjamini, Krieger and Yekutieli | Predicted (LS) mean diff. | Discovery | P-Value |
| --- | --- | --- | --- |
| 450 | -0.05713 | No | 0.9698 |
| 455 | 0.2006 | No | 0.8943 |
| 460 | -0.3632 | No | 0.8099 |
| 465 | -0.8107 | No | 0.5914 |
| 470 | -0.9885 | No | 0.5128 |
| 475 | -0.2639 | No | 0.8613 |
| 480 | 0.3462 | No | 0.8187 |
| 485 | 0.2037 | No | 0.8927 |
| 490 | -0.5053 | No | 0.7379 |
| 495 | -1.361 | No | 0.3676 |
| 500 | -1.272 | No | 0.3999 |
| 505 | -0.8240 | No | 0.5854 |
| 510 | -0.3497 | No | 0.8169 |
| 515 | -0.2229 | No | 0.8826 |
| 520 | -0.7779 | No | 0.6065 |
| 525 | -1.673 | No | 0.2682 |
| 530 | -2.004 | No | 0.1847 |
| 535 | -1.698 | No | 0.2611 |
| 540 | -1.347 | No | 0.3726 |
| 545 | -1.155 | No | 0.4445 |
| 550 | -1.300 | No | 0.3894 |
| 555 | -1.799 | No | 0.2337 |
| 560 | -2.282 | No | 0.1309 |
| 565 | -2.825 | No | 0.0616 |
| 570 | -2.416 | No | 0.1098 |
| 575 | -1.653 | No | 0.2739 |
| 580 | -1.178 | No | 0.4353 |
| 585 | -0.7378 | No | 0.6252 |
| 590 | -0.7482 | No | 0.6203 |
| 595 | -0.7820 | No | 0.6046 |
| 600 | -1.171 | No | 0.4380 |
| 605 | -1.114 | No | 0.4606 |
| 610 | -1.346 | No | 0.3730 |
| 615 | -1.460 | No | 0.3338 |
| 620 | -1.010 | No | 0.5037 |
| 625 | -0.4104 | No | 0.7858 |
| 630 | 0.08889 | No | 0.9531 |
| 635 | 0.3629 | No | 0.8101 |
| 640 | 0.5322 | No | 0.7246 |
| 645 | 0.4710 | No | 0.7551 |
| 650 | -0.2155 | No | 0.8865 |
| 655 | -0.4516 | No | 0.7649 |
| 660 | -0.7879 | No | 0.6019 |
| 665 | -0.8552 | No | 0.5712 |
| 670 | -0.3988 | No | 0.7917 |
| 675 | -0.1604 | No | 0.9154 |
| 680 | 0.1190 | No | 0.9372 |

**Supplementary Table 3. Statistics for 14-month-old WT vs A53T mice**

Two-way repeated measures ANOVA where, DF (degrees of freedom), SS (sum of squares), MS (mean square), and post-hoc comparisons between 14-month-old WT and A53T mice **(A)** % reflectance, and **(B)** residual

| 1. **% reflectance** | SS | DF | MS | F (DFn, DFd) | P-value |
| --- | --- | --- | --- | --- | --- |
| Wavelength x Genotype | 1073 | 46 | 23.32 | F (46, 1426) = 2.795 | P<0.0001 |
| Wavelength | 127484 | 46 | 2771 | F (46, 1426) = 332.1 | P<0.0001 |
| Genotype | 2857 | 1 | 2857 | F (1, 31) = 7.039 | P=0.0125 |
| Subject | 12582 | 31 | 405.9 | F (31, 1426) = 48.64 | P<0.0001 |
| Residual | 11899 | 1426 | 8.344 |  |  |

| Two-stage linear step-up procedure of Benjamini, Krieger and Yekutieli | Predicted (LS) mean diff. | Discovery | P-Value |
| --- | --- | --- | --- |
| 450 | -3.347 | Yes | 0.0197 |
| 455 | -3.581 | Yes | 0.0126 |
| 460 | -4.294 | Yes | 0.0028 |
| 465 | -4.853 | Yes | 0.0007 |
| 470 | -4.913 | Yes | 0.0006 |
| 475 | -5.012 | Yes | 0.0005 |
| 480 | -4.783 | Yes | 0.0009 |
| 485 | -4.559 | Yes | 0.0015 |
| 490 | -4.659 | Yes | 0.0012 |
| 495 | -4.472 | Yes | 0.0018 |
| 500 | -4.769 | Yes | 0.0009 |
| 505 | -4.372 | Yes | 0.0023 |
| 510 | -3.955 | Yes | 0.0059 |
| 515 | -3.928 | Yes | 0.0062 |
| 520 | -3.686 | Yes | 0.0102 |
| 525 | -3.848 | Yes | 0.0073 |
| 530 | -3.976 | Yes | 0.0056 |
| 535 | -4.012 | Yes | 0.0052 |
| 540 | -4.152 | Yes | 0.0038 |
| 545 | -3.467 | Yes | 0.0157 |
| 550 | -3.021 | Yes | 0.0352 |
| 555 | -2.762 | Yes | 0.0541 |
| 560 | -2.861 | Yes | 0.0461 |
| 565 | -3.046 | Yes | 0.0337 |
| 570 | -3.538 | Yes | 0.0137 |
| 575 | -3.639 | Yes | 0.0112 |
| 580 | -3.648 | Yes | 0.0110 |
| 585 | -2.795 | Yes | 0.0513 |
| 590 | -2.395 | No | 0.0949 |
| 595 | -1.805 | No | 0.2080 |
| 600 | -1.686 | No | 0.2396 |
| 605 | -1.980 | No | 0.1673 |
| 610 | -1.791 | No | 0.2115 |
| 615 | -1.979 | No | 0.1675 |
| 620 | -2.293 | No | 0.1098 |
| 625 | -1.578 | No | 0.2710 |
| 630 | -0.9999 | No | 0.4855 |
| 635 | -0.5569 | No | 0.6976 |
| 640 | 0.3376 | No | 0.8138 |
| 645 | 0.4081 | No | 0.7758 |
| 650 | 1.305 | No | 0.3625 |
| 655 | -0.1117 | No | 0.9379 |
| 660 | -0.3825 | No | 0.7896 |
| 665 | -0.5347 | No | 0.7091 |
| 670 | -1.031 | No | 0.4721 |
| 675 | -0.4914 | No | 0.7317 |
| 680 | -0.5902 | No | 0.6805 |

| 1. **residual** | SS | DF | MS | F (DFn, DFd) | P-value | |
| --- | --- | --- | --- | --- | --- | --- |
| Wavelength x Genotype | 1073 | 46 | 23.32 | F (46, 1426) = 2.795 | P<0.0001 |  |
| Wavelength | 1073 | 46 | 23.32 | F (46, 1426) = 2.795 | P<0.0001 |  |
| Genotype | 2857 | 1 | 2857 | F (1, 31) = 7.039 | P=0.0125 |  |
| Subject | 12582 | 31 | 405.9 | F (31, 1426) = 48.64 | P<0.0001 |  |
| Residual | 11899 | 1426 | 8.344 |  |  |  |

| Two-stage linear step-up procedure of Benjamini, Krieger and Yekutieli | Predicted (LS) mean diff. | Discovery | P-Value |
| --- | --- | --- | --- |
| 450 | -3.347 | Yes | 0.0197 |
| 455 | -3.581 | Yes | 0.0126 |
| 460 | -4.294 | Yes | 0.0028 |
| 465 | -4.853 | Yes | 0.0007 |
| 470 | -4.913 | Yes | 0.0006 |
| 475 | -5.012 | Yes | 0.0005 |
| 480 | -4.783 | Yes | 0.0009 |
| 485 | -4.559 | Yes | 0.0015 |
| 490 | -4.659 | Yes | 0.0012 |
| 495 | -4.472 | Yes | 0.0018 |
| 500 | -4.769 | Yes | 0.0009 |
| 505 | -4.372 | Yes | 0.0023 |
| 510 | -3.955 | Yes | 0.0059 |
| 515 | -3.928 | Yes | 0.0062 |
| 520 | -3.686 | Yes | 0.0102 |
| 525 | -3.848 | Yes | 0.0073 |
| 530 | -3.976 | Yes | 0.0056 |
| 535 | -4.012 | Yes | 0.0052 |
| 540 | -4.152 | Yes | 0.0038 |
| 545 | -3.467 | Yes | 0.0157 |
| 550 | -3.021 | Yes | 0.0352 |
| 555 | -2.762 | Yes | 0.0541 |
| 560 | -2.861 | Yes | 0.0461 |
| 565 | -3.046 | Yes | 0.0337 |
| 570 | -3.538 | Yes | 0.0137 |
| 575 | -3.639 | Yes | 0.0112 |
| 580 | -3.648 | Yes | 0.0110 |
| 585 | -2.795 | Yes | 0.0513 |
| 590 | -2.395 | No | 0.0949 |
| 595 | -1.805 | No | 0.2080 |
| 600 | -1.686 | No | 0.2396 |
| 605 | -1.980 | No | 0.1673 |
| 610 | -1.791 | No | 0.2115 |
| 615 | -1.979 | No | 0.1675 |
| 620 | -2.293 | No | 0.1098 |
| 625 | -1.578 | No | 0.2710 |
| 630 | -0.9999 | No | 0.4855 |
| 635 | -0.5569 | No | 0.6976 |
| 640 | 0.3376 | No | 0.8138 |
| 645 | 0.4081 | No | 0.7758 |
| 650 | 1.305 | No | 0.3625 |
| 655 | -0.1117 | No | 0.9379 |
| 660 | -0.3825 | No | 0.7896 |
| 665 | -0.5347 | No | 0.7091 |
| 670 | -1.031 | No | 0.4721 |
| 675 | -0.4914 | No | 0.7317 |
| 680 | -0.5902 | No | 0.6805 |

**Supplementary Table 4. Statistics for 8-month-old WT vs TauKO mice**

Two-way repeated measures ANOVA where, DF (degrees of freedom), SS (sum of squares), MS (mean square), and post-hoc comparisons between 8-month-old WT and TauKO mice **(A)** % reflectance, and **(B)** residual

| 1. **% reflectance** | SS | DF | MS | F (DFn, DFd) | P-value |
| --- | --- | --- | --- | --- | --- |
| Wavelength x Genotype | 159.7 | 46 | 3.471 | F (46, 966) = 0.5839 | P=0.9881 |
| Wavelength | 191460 | 46 | 4162 | F (46, 966) = 700.2 | P<0.0001 |
| Genotype | 0.5026 | 1 | 0.5026 | F (1, 21) = 0.002056 | P=0.9643 |
| Subject | 5134 | 21 | 244.5 | F (21, 966) = 41.13 | P<0.0001 |
| Residual | 5742 | 966 | 5.944 |  |  |

| Two-stage linear step-up procedure of Benjamini, Krieger and Yekutieli | Predicted (LS) mean diff. | Discovery | P-Value |
| --- | --- | --- | --- |
| 450 | -1.090 | No | 0.4351 |
| 455 | -0.9678 | No | 0.4884 |
| 460 | -1.307 | No | 0.3496 |
| 465 | -1.425 | No | 0.3078 |
| 470 | 0.02963 | No | 0.9831 |
| 475 | 0.4192 | No | 0.7640 |
| 480 | -0.3380 | No | 0.8087 |
| 485 | -0.07568 | No | 0.9568 |
| 490 | -0.4781 | No | 0.7321 |
| 495 | -0.06110 | No | 0.9651 |
| 500 | 0.8931 | No | 0.5225 |
| 505 | 1.435 | No | 0.3043 |
| 510 | 1.385 | No | 0.3214 |
| 515 | 0.5384 | No | 0.6999 |
| 520 | -0.1924 | No | 0.8904 |
| 525 | -0.2925 | No | 0.8341 |
| 530 | 0.01786 | No | 0.9898 |
| 535 | 0.6115 | No | 0.6615 |
| 540 | 1.099 | No | 0.4315 |
| 545 | 1.427 | No | 0.3070 |
| 550 | 0.5707 | No | 0.6828 |
| 555 | 0.04839 | No | 0.9724 |
| 560 | -0.2864 | No | 0.8375 |
| 565 | -0.2855 | No | 0.8380 |
| 570 | 0.2529 | No | 0.8563 |
| 575 | 0.4389 | No | 0.7533 |
| 580 | 0.6130 | No | 0.6607 |
| 585 | 0.3455 | No | 0.8046 |
| 590 | 1.242 | No | 0.3741 |
| 595 | -0.2212 | No | 0.8741 |
| 600 | -0.4075 | No | 0.7704 |
| 605 | -0.5853 | No | 0.6752 |
| 610 | -0.7927 | No | 0.5704 |
| 615 | -0.2644 | No | 0.8498 |
| 620 | 0.8021 | No | 0.5658 |
| 625 | 0.8255 | No | 0.5545 |
| 630 | 1.226 | No | 0.3800 |
| 635 | 1.190 | No | 0.3943 |
| 640 | 0.05688 | No | 0.9675 |
| 645 | 0.2354 | No | 0.8662 |
| 650 | -0.8814 | No | 0.5280 |
| 655 | -1.207 | No | 0.3877 |
| 660 | -0.6202 | No | 0.6570 |
| 665 | -0.8789 | No | 0.5292 |
| 670 | -1.087 | No | 0.4364 |
| 675 | -0.2873 | No | 0.8370 |
| 680 | 0.3731 | No | 0.7894 |

| 1. **residual** | SS | DF | MS | F (DFn, DFd) | P-value |
| --- | --- | --- | --- | --- | --- |
| Wavelength x Genotype | 159.7 | 46 | 3.471 | F (46, 966) = 0.5839 | P=0.9881 |
| Wavelength | 159.7 | 46 | 3.471 | F (46, 966) = 0.5839 | P=0.9881 |
| Genotype | 0.5026 | 1 | 0.5026 | F (1, 21) = 0.002056 | P=0.9643 |
| Subject | 5134 | 21 | 244.5 | F (21, 966) = 41.13 | P<0.0001 |
| Residual | 5742 | 966 | 5.944 |  |  |

| Two-stage linear step-up procedure of Benjamini, Krieger and Yekutieli | Predicted (LS) mean diff. | Discovery | P-Value |
| --- | --- | --- | --- |
| 450 | -1.090 | No | 0.4351 |
| 455 | -0.9678 | No | 0.4884 |
| 460 | -1.307 | No | 0.3496 |
| 465 | -1.425 | No | 0.3078 |
| 470 | 0.02963 | No | 0.9831 |
| 475 | 0.4192 | No | 0.7640 |
| 480 | -0.3380 | No | 0.8087 |
| 485 | -0.07568 | No | 0.9568 |
| 490 | -0.4781 | No | 0.7321 |
| 495 | -0.06110 | No | 0.9651 |
| 500 | 0.8931 | No | 0.5225 |
| 505 | 1.435 | No | 0.3043 |
| 510 | 1.385 | No | 0.3214 |
| 515 | 0.5384 | No | 0.6999 |
| 520 | -0.1924 | No | 0.8904 |
| 525 | -0.2925 | No | 0.8341 |
| 530 | 0.01786 | No | 0.9898 |
| 535 | 0.6115 | No | 0.6615 |
| 540 | 1.099 | No | 0.4315 |
| 545 | 1.427 | No | 0.3070 |
| 550 | 0.5707 | No | 0.6828 |
| 555 | 0.04839 | No | 0.9724 |
| 560 | -0.2864 | No | 0.8375 |
| 565 | -0.2855 | No | 0.8380 |
| 570 | 0.2529 | No | 0.8563 |
| 575 | 0.4389 | No | 0.7533 |
| 580 | 0.6130 | No | 0.6607 |
| 585 | 0.3455 | No | 0.8046 |
| 590 | 1.242 | No | 0.3741 |
| 595 | -0.2212 | No | 0.8741 |
| 600 | -0.4075 | No | 0.7704 |
| 605 | -0.5853 | No | 0.6752 |
| 610 | -0.7927 | No | 0.5704 |
| 615 | -0.2644 | No | 0.8498 |
| 620 | 0.8021 | No | 0.5658 |
| 625 | 0.8255 | No | 0.5545 |
| 630 | 1.226 | No | 0.3800 |
| 635 | 1.190 | No | 0.3943 |
| 640 | 0.05688 | No | 0.9675 |
| 645 | 0.2354 | No | 0.8662 |
| 650 | -0.8814 | No | 0.5280 |
| 655 | -1.207 | No | 0.3877 |
| 660 | -0.6202 | No | 0.6570 |
| 665 | -0.8789 | No | 0.5292 |
| 670 | -1.087 | No | 0.4364 |
| 675 | -0.2873 | No | 0.8370 |
| 680 | 0.3731 | No | 0.7894 |

**Supplementary Table 5. Statistics for 18-month-old WT vs TauKO mice**

Two-way repeated measures ANOVA where, DF (degrees of freedom), SS (sum of squares), MS (mean square), and post-hoc comparisons between 18-month-old WT and TauKO mice **(A)** % reflectance, and **(B)** residual

| 1. **% reflectance** | SS | DF | MS | F (DFn, DFd) | P-value |
| --- | --- | --- | --- | --- | --- |
| Wavelength x Genotype | 1291 | 46 | 28.07 | F (46, 1288) = 3.510 | P<0.0001 |
| Wavelength | 269040 | 46 | 5849 | F (46, 1288) = 731.3 | P<0.0001 |
| Genotype | 2370 | 1 | 2370 | F (1, 28) = 6.759 | P=0.0147 |
| Subject | 9819 | 28 | 350.7 | F (28, 1288) = 43.85 | P<0.0001 |
| Residual | 10302 | 1288 | 7.998 |  |  |

| Two-stage linear step-up procedure of Benjamini, Krieger and Yekutieli | Predicted (LS) mean diff. | Discovery | P-Value |
| --- | --- | --- | --- |
| 450 | -4.581 | Yes | 0.0014 |
| 455 | -4.340 | Yes | 0.0025 |
| 460 | -5.329 | Yes | 0.0002 |
| 465 | -6.474 | Yes | <0.0001 |
| 470 | -5.796 | Yes | <0.0001 |
| 475 | -4.988 | Yes | 0.0005 |
| 480 | -3.316 | Yes | 0.0206 |
| 485 | -3.325 | Yes | 0.0203 |
| 490 | -3.912 | Yes | 0.0063 |
| 495 | -4.291 | Yes | 0.0028 |
| 500 | -2.947 | No | 0.0397 |
| 505 | -2.991 | No | 0.0368 |
| 510 | -2.763 | No | 0.0537 |
| 515 | -2.127 | No | 0.1374 |
| 520 | -2.870 | No | 0.0451 |
| 525 | -3.776 | Yes | 0.0084 |
| 530 | -4.546 | Yes | 0.0015 |
| 535 | -3.775 | Yes | 0.0084 |
| 540 | -3.817 | Yes | 0.0077 |
| 545 | -3.074 | Yes | 0.0319 |
| 550 | -2.745 | No | 0.0553 |
| 555 | -3.309 | Yes | 0.0209 |
| 560 | -3.850 | Yes | 0.0072 |
| 565 | -4.727 | Yes | 0.0010 |
| 570 | -5.150 | Yes | 0.0003 |
| 575 | -5.008 | Yes | 0.0005 |
| 580 | -3.562 | Yes | 0.0129 |
| 585 | -2.586 | No | 0.0710 |
| 590 | -1.982 | No | 0.1663 |
| 595 | -1.366 | No | 0.3399 |
| 600 | -1.443 | No | 0.3133 |
| 605 | -1.775 | No | 0.2150 |
| 610 | -1.557 | No | 0.2768 |
| 615 | -1.134 | No | 0.4284 |
| 620 | -0.7094 | No | 0.6202 |
| 625 | 0.7701 | No | 0.5905 |
| 630 | 1.035 | No | 0.4695 |
| 635 | 1.134 | No | 0.4281 |
| 640 | 0.8682 | No | 0.5442 |
| 645 | 0.5597 | No | 0.6958 |
| 650 | -0.7201 | No | 0.6149 |
| 655 | -1.157 | No | 0.4189 |
| 660 | -1.720 | No | 0.2295 |
| 665 | -1.330 | No | 0.3528 |
| 670 | -1.517 | No | 0.2894 |
| 675 | -0.1338 | No | 0.9255 |
| 680 | 0.0008358 | No | 0.9995 |

| 1. **residual** | SS | DF | MS | F (DFn, DFd) | P-value |
| --- | --- | --- | --- | --- | --- |
| Wavelength x Genotype | 1291 | 46 | 28.07 | F (46, 1288) = 3.510 | P<0.0001 |
| Wavelength | 1291 | 46 | 28.07 | F (46, 1288) = 3.510 | P<0.0001 |
| Genotype | 2370 | 1 | 2370 | F (1, 28) = 6.759 | P=0.0147 |
| Subject | 9819 | 28 | 350.7 | F (28, 1288) = 43.85 | P<0.0001 |
| Residual | 10302 | 1288 | 7.998 |  |  |

| Two-stage linear step-up procedure of Benjamini, Krieger and Yekutieli | Predicted (LS) mean diff. | Discovery | P-Value |
| --- | --- | --- | --- |
| 450 | -4.581 | Yes | 0.0014 |
| 455 | -4.340 | Yes | 0.0025 |
| 460 | -5.329 | Yes | 0.0002 |
| 465 | -6.474 | Yes | <0.0001 |
| 470 | -5.796 | Yes | <0.0001 |
| 475 | -4.988 | Yes | 0.0005 |
| 480 | -3.316 | Yes | 0.0206 |
| 485 | -3.325 | Yes | 0.0203 |
| 490 | -3.912 | Yes | 0.0063 |
| 495 | -4.291 | Yes | 0.0028 |
| 500 | -2.947 | No | 0.0397 |
| 505 | -2.991 | No | 0.0368 |
| 510 | -2.763 | No | 0.0537 |
| 515 | -2.127 | No | 0.1374 |
| 520 | -2.870 | No | 0.0451 |
| 525 | -3.776 | Yes | 0.0084 |
| 530 | -4.546 | Yes | 0.0015 |
| 535 | -3.775 | Yes | 0.0084 |
| 540 | -3.817 | Yes | 0.0077 |
| 545 | -3.074 | Yes | 0.0319 |
| 550 | -2.745 | No | 0.0553 |
| 555 | -3.309 | Yes | 0.0209 |
| 560 | -3.850 | Yes | 0.0072 |
| 565 | -4.727 | Yes | 0.0010 |
| 570 | -5.150 | Yes | 0.0003 |
| 575 | -5.008 | Yes | 0.0005 |
| 580 | -3.562 | Yes | 0.0129 |
| 585 | -2.586 | No | 0.0710 |
| 590 | -1.982 | No | 0.1663 |
| 595 | -1.366 | No | 0.3399 |
| 600 | -1.443 | No | 0.3133 |
| 605 | -1.775 | No | 0.2150 |
| 610 | -1.557 | No | 0.2768 |
| 615 | -1.134 | No | 0.4284 |
| 620 | -0.7094 | No | 0.6202 |
| 625 | 0.7701 | No | 0.5905 |
| 630 | 1.035 | No | 0.4695 |
| 635 | 1.134 | No | 0.4281 |
| 640 | 0.8682 | No | 0.5442 |
| 645 | 0.5597 | No | 0.6958 |
| 650 | -0.7201 | No | 0.6149 |
| 655 | -1.157 | No | 0.4189 |
| 660 | -1.720 | No | 0.2295 |
| 665 | -1.330 | No | 0.3528 |
| 670 | -1.517 | No | 0.2894 |
| 675 | -0.1338 | No | 0.9255 |
| 680 | 0.0008359 | No | 0.9995 |

**Supplementary Table 6. Statistics for C57blk6J mice**

Two-way repeated measures ANOVA where, DF (degrees of freedom), SS (sum of squares), MS (mean square), and post-hoc comparisons of normal ageing in C57blk6J mice **(A)** % reflectance, and **(B)** residual

| 1. **% reflectance** | SS | DF | MS | F (DFn, DFd) | P-value |
| --- | --- | --- | --- | --- | --- |
| Wavelength x Age | 19351 | 92 | 210.3 | F (92, 1058) = 11.63 | P<0.0001 |
| Wavelength | 188679 | 46 | 4102 | F (46, 1058) = 226.8 | P<0.0001 |
| Age | 33948 | 2 | 16974 | F (2, 23) = 35.61 | P<0.0001 |
| Subject | 10963 | 23 | 476.6 | F (23, 1058) = 26.36 | P<0.0001 |

| Two-stage linear step-up procedure of Benjamini, Krieger and Yekutieli | Predicted (LS) mean diff. | Discovery | P-Value |
| --- | --- | --- | --- |
|  |  |  |  |
| 450 |  |  |  |
| 3mo C57blk6J vs. 6mo C57blk6J | 2.072 | No | 0.4051 |
| 3mo C57blk6J vs. 12mo C57blk6J | 14.46 | Yes | <0.0001 |
| 6mo C57blk6J vs. 12mo C57blk6J | 12.39 | Yes | <0.0001 |
|  |  |  |  |
| 455 |  |  |  |
| 3mo C57blk6J vs. 6mo C57blk6J | 0.8178 | No | 0.7424 |
| 3mo C57blk6J vs. 12mo C57blk6J | 15.07 | Yes | <0.0001 |
| 6mo C57blk6J vs. 12mo C57blk6J | 14.25 | Yes | <0.0001 |
|  |  |  |  |
| 460 |  |  |  |
| 3mo C57blk6J vs. 6mo C57blk6J | 5.591 | Yes | 0.0248 |
| 3mo C57blk6J vs. 12mo C57blk6J | 20.93 | Yes | <0.0001 |
| 6mo C57blk6J vs. 12mo C57blk6J | 15.34 | Yes | <0.0001 |
|  |  |  |  |
| 465 |  |  |  |
| 3mo C57blk6J vs. 6mo C57blk6J | 12.85 | Yes | <0.0001 |
| 3mo C57blk6J vs. 12mo C57blk6J | 27.63 | Yes | <0.0001 |
| 6mo C57blk6J vs. 12mo C57blk6J | 14.78 | Yes | <0.0001 |
|  |  |  |  |
| 470 |  |  |  |
| 3mo C57blk6J vs. 6mo C57blk6J | 14.83 | Yes | <0.0001 |
| 3mo C57blk6J vs. 12mo C57blk6J | 29.15 | Yes | <0.0001 |
| 6mo C57blk6J vs. 12mo C57blk6J | 14.32 | Yes | <0.0001 |
|  |  |  |  |
| 475 |  |  |  |
| 3mo C57blk6J vs. 6mo C57blk6J | 9.386 | Yes | 0.0002 |
| 3mo C57blk6J vs. 12mo C57blk6J | 23.89 | Yes | <0.0001 |
| 6mo C57blk6J vs. 12mo C57blk6J | 14.50 | Yes | <0.0001 |
|  |  |  |  |
| 480 |  |  |  |
| 3mo C57blk6J vs. 6mo C57blk6J | 1.248 | No | 0.6160 |
| 3mo C57blk6J vs. 12mo C57blk6J | 17.37 | Yes | <0.0001 |
| 6mo C57blk6J vs. 12mo C57blk6J | 16.13 | Yes | <0.0001 |
|  |  |  |  |
| 485 |  |  |  |
| 3mo C57blk6J vs. 6mo C57blk6J | 0.09790 | No | 0.9686 |
| 3mo C57blk6J vs. 12mo C57blk6J | 17.18 | Yes | <0.0001 |
| 6mo C57blk6J vs. 12mo C57blk6J | 17.08 | Yes | <0.0001 |
|  |  |  |  |
| 490 |  |  |  |
| 3mo C57blk6J vs. 6mo C57blk6J | 6.167 | Yes | 0.0133 |
| 3mo C57blk6J vs. 12mo C57blk6J | 21.61 | Yes | <0.0001 |
| 6mo C57blk6J vs. 12mo C57blk6J | 15.44 | Yes | <0.0001 |
|  |  |  |  |
| 495 |  |  |  |
| 3mo C57blk6J vs. 6mo C57blk6J | 13.16 | Yes | <0.0001 |
| 3mo C57blk6J vs. 12mo C57blk6J | 27.27 | Yes | <0.0001 |
| 6mo C57blk6J vs. 12mo C57blk6J | 14.11 | Yes | <0.0001 |
|  |  |  |  |
| 500 |  |  |  |
| 3mo C57blk6J vs. 6mo C57blk6J | 14.44 | Yes | <0.0001 |
| 3mo C57blk6J vs. 12mo C57blk6J | 26.72 | Yes | <0.0001 |
| 6mo C57blk6J vs. 12mo C57blk6J | 12.28 | Yes | <0.0001 |
|  |  |  |  |
| 505 |  |  |  |
| 3mo C57blk6J vs. 6mo C57blk6J | 9.264 | Yes | 0.0002 |
| 3mo C57blk6J vs. 12mo C57blk6J | 21.65 | Yes | <0.0001 |
| 6mo C57blk6J vs. 12mo C57blk6J | 12.39 | Yes | <0.0001 |
|  |  |  |  |
| 510 |  |  |  |
| 3mo C57blk6J vs. 6mo C57blk6J | 2.014 | No | 0.4183 |
| 3mo C57blk6J vs. 12mo C57blk6J | 15.86 | Yes | <0.0001 |
| 6mo C57blk6J vs. 12mo C57blk6J | 13.84 | Yes | <0.0001 |
|  |  |  |  |
| 515 |  |  |  |
| 3mo C57blk6J vs. 6mo C57blk6J | -1.566 | No | 0.5292 |
| 3mo C57blk6J vs. 12mo C57blk6J | 12.53 | Yes | <0.0001 |
| 6mo C57blk6J vs. 12mo C57blk6J | 14.09 | Yes | <0.0001 |
|  |  |  |  |
| 520 |  |  |  |
| 3mo C57blk6J vs. 6mo C57blk6J | 1.171 | No | 0.6379 |
| 3mo C57blk6J vs. 12mo C57blk6J | 13.86 | Yes | <0.0001 |
| 6mo C57blk6J vs. 12mo C57blk6J | 12.69 | Yes | <0.0001 |
|  |  |  |  |
| 525 |  |  |  |
| 3mo C57blk6J vs. 6mo C57blk6J | 8.124 | Yes | 0.0011 |
| 3mo C57blk6J vs. 12mo C57blk6J | 19.49 | Yes | <0.0001 |
| 6mo C57blk6J vs. 12mo C57blk6J | 11.37 | Yes | <0.0001 |
|  |  |  |  |
| 530 |  |  |  |
| 3mo C57blk6J vs. 6mo C57blk6J | 13.80 | Yes | <0.0001 |
| 3mo C57blk6J vs. 12mo C57blk6J | 23.12 | Yes | <0.0001 |
| 6mo C57blk6J vs. 12mo C57blk6J | 9.315 | Yes | 0.0003 |
|  |  |  |  |
| 535 |  |  |  |
| 3mo C57blk6J vs. 6mo C57blk6J | 13.36 | Yes | <0.0001 |
| 3mo C57blk6J vs. 12mo C57blk6J | 23.12 | Yes | <0.0001 |
| 6mo C57blk6J vs. 12mo C57blk6J | 9.759 | Yes | 0.0001 |
|  |  |  |  |
| 540 |  |  |  |
| 3mo C57blk6J vs. 6mo C57blk6J | 8.182 | Yes | 0.0010 |
| 3mo C57blk6J vs. 12mo C57blk6J | 18.36 | Yes | <0.0001 |
| 6mo C57blk6J vs. 12mo C57blk6J | 10.18 | Yes | <0.0001 |
|  |  |  |  |
| 545 |  |  |  |
| 3mo C57blk6J vs. 6mo C57blk6J | 2.223 | No | 0.3717 |
| 3mo C57blk6J vs. 12mo C57blk6J | 13.43 | Yes | <0.0001 |
| 6mo C57blk6J vs. 12mo C57blk6J | 11.21 | Yes | <0.0001 |
|  |  |  |  |
| 550 |  |  |  |
| 3mo C57blk6J vs. 6mo C57blk6J | -2.748 | No | 0.2695 |
| 3mo C57blk6J vs. 12mo C57blk6J | 8.329 | Yes | 0.0012 |
| 6mo C57blk6J vs. 12mo C57blk6J | 11.08 | Yes | <0.0001 |
|  |  |  |  |
| 555 |  |  |  |
| 3mo C57blk6J vs. 6mo C57blk6J | -2.712 | No | 0.2758 |
| 3mo C57blk6J vs. 12mo C57blk6J | 7.980 | Yes | 0.0019 |
| 6mo C57blk6J vs. 12mo C57blk6J | 10.69 | Yes | <0.0001 |
|  |  |  |  |
| 560 |  |  |  |
| 3mo C57blk6J vs. 6mo C57blk6J | 2.318 | No | 0.3516 |
| 3mo C57blk6J vs. 12mo C57blk6J | 11.66 | Yes | <0.0001 |
| 6mo C57blk6J vs. 12mo C57blk6J | 9.345 | Yes | 0.0003 |
|  |  |  |  |
| 565 |  |  |  |
| 3mo C57blk6J vs. 6mo C57blk6J | 9.601 | Yes | 0.0001 |
| 3mo C57blk6J vs. 12mo C57blk6J | 16.32 | Yes | <0.0001 |
| 6mo C57blk6J vs. 12mo C57blk6J | 6.718 | Yes | 0.0089 |
|  |  |  |  |
| 570 |  |  |  |
| 3mo C57blk6J vs. 6mo C57blk6J | 13.42 | Yes | <0.0001 |
| 3mo C57blk6J vs. 12mo C57blk6J | 19.07 | Yes | <0.0001 |
| 6mo C57blk6J vs. 12mo C57blk6J | 5.650 | Yes | 0.0277 |
|  |  |  |  |
| 575 |  |  |  |
| 3mo C57blk6J vs. 6mo C57blk6J | 11.54 | Yes | <0.0001 |
| 3mo C57blk6J vs. 12mo C57blk6J | 17.80 | Yes | <0.0001 |
| 6mo C57blk6J vs. 12mo C57blk6J | 6.256 | Yes | 0.0148 |
|  |  |  |  |
| 580 |  |  |  |
| 3mo C57blk6J vs. 6mo C57blk6J | 7.417 | Yes | 0.0029 |
| 3mo C57blk6J vs. 12mo C57blk6J | 14.73 | Yes | <0.0001 |
| 6mo C57blk6J vs. 12mo C57blk6J | 7.312 | Yes | 0.0044 |
|  |  |  |  |
| 585 |  |  |  |
| 3mo C57blk6J vs. 6mo C57blk6J | 2.360 | No | 0.3429 |
| 3mo C57blk6J vs. 12mo C57blk6J | 9.889 | Yes | 0.0001 |
| 6mo C57blk6J vs. 12mo C57blk6J | 7.529 | Yes | 0.0034 |
|  |  |  |  |
| 590 |  |  |  |
| 3mo C57blk6J vs. 6mo C57blk6J | -2.143 | No | 0.3891 |
| 3mo C57blk6J vs. 12mo C57blk6J | 5.399 | Yes | 0.0355 |
| 6mo C57blk6J vs. 12mo C57blk6J | 7.542 | Yes | 0.0033 |
|  |  |  |  |
| 595 |  |  |  |
| 3mo C57blk6J vs. 6mo C57blk6J | -3.442 | No | 0.1667 |
| 3mo C57blk6J vs. 12mo C57blk6J | 2.754 | No | 0.2830 |
| 6mo C57blk6J vs. 12mo C57blk6J | 6.196 | Yes | 0.0158 |
|  |  |  |  |
| 600 |  |  |  |
| 3mo C57blk6J vs. 6mo C57blk6J | -0.03413 | No | 0.9891 |
| 3mo C57blk6J vs. 12mo C57blk6J | 3.743 | No | 0.1446 |
| 6mo C57blk6J vs. 12mo C57blk6J | 3.778 | No | 0.1410 |
|  |  |  |  |
| 605 |  |  |  |
| 3mo C57blk6J vs. 6mo C57blk6J | 5.303 | Yes | 0.0332 |
| 3mo C57blk6J vs. 12mo C57blk6J | 7.801 | Yes | 0.0024 |
| 6mo C57blk6J vs. 12mo C57blk6J | 2.498 | No | 0.3301 |
|  |  |  |  |
| 610 |  |  |  |
| 3mo C57blk6J vs. 6mo C57blk6J | 11.88 | Yes | <0.0001 |
| 3mo C57blk6J vs. 12mo C57blk6J | 13.89 | Yes | <0.0001 |
| 6mo C57blk6J vs. 12mo C57blk6J | 2.004 | No | 0.4346 |
|  |  |  |  |
| 615 |  |  |  |
| 3mo C57blk6J vs. 6mo C57blk6J | 13.62 | Yes | <0.0001 |
| 3mo C57blk6J vs. 12mo C57blk6J | 15.83 | Yes | <0.0001 |
| 6mo C57blk6J vs. 12mo C57blk6J | 2.212 | No | 0.3884 |
|  |  |  |  |
| 620 |  |  |  |
| 3mo C57blk6J vs. 6mo C57blk6J | 11.38 | Yes | <0.0001 |
| 3mo C57blk6J vs. 12mo C57blk6J | 14.83 | Yes | <0.0001 |
| 6mo C57blk6J vs. 12mo C57blk6J | 3.451 | No | 0.1786 |
|  |  |  |  |
| 625 |  |  |  |
| 3mo C57blk6J vs. 6mo C57blk6J | 7.546 | Yes | 0.0025 |
| 3mo C57blk6J vs. 12mo C57blk6J | 11.74 | Yes | <0.0001 |
| 6mo C57blk6J vs. 12mo C57blk6J | 4.194 | Yes | 0.1022 |
|  |  |  |  |
| 630 |  |  |  |
| 3mo C57blk6J vs. 6mo C57blk6J | 2.824 | No | 0.2566 |
| 3mo C57blk6J vs. 12mo C57blk6J | 7.643 | Yes | 0.0029 |
| 6mo C57blk6J vs. 12mo C57blk6J | 4.819 | No | 0.0604 |
|  |  |  |  |
| 635 |  |  |  |
| 3mo C57blk6J vs. 6mo C57blk6J | -2.108 | No | 0.3968 |
| 3mo C57blk6J vs. 12mo C57blk6J | 2.123 | No | 0.4078 |
| 6mo C57blk6J vs. 12mo C57blk6J | 4.232 | No | 0.0991 |
|  |  |  |  |
| 640 |  |  |  |
| 3mo C57blk6J vs. 6mo C57blk6J | -5.504 | No | 0.0271 |
| 3mo C57blk6J vs. 12mo C57blk6J | -2.793 | No | 0.2762 |
| 6mo C57blk6J vs. 12mo C57blk6J | 2.711 | No | 0.2907 |
|  |  |  |  |
| 645 |  |  |  |
| 3mo C57blk6J vs. 6mo C57blk6J | -6.151 | Yes | 0.0136 |
| 3mo C57blk6J vs. 12mo C57blk6J | -5.506 | Yes | 0.0320 |
| 6mo C57blk6J vs. 12mo C57blk6J | 0.6449 | No | 0.8015 |
|  |  |  |  |
| 650 |  |  |  |
| 3mo C57blk6J vs. 6mo C57blk6J | -1.945 | No | 0.4344 |
| 3mo C57blk6J vs. 12mo C57blk6J | -5.078 | No | 0.0479 |
| 6mo C57blk6J vs. 12mo C57blk6J | -3.133 | No | 0.2220 |
|  |  |  |  |
| 655 |  |  |  |
| 3mo C57blk6J vs. 6mo C57blk6J | 4.034 | No | 0.1052 |
| 3mo C57blk6J vs. 12mo C57blk6J | -0.4453 | No | 0.8621 |
| 6mo C57blk6J vs. 12mo C57blk6J | -4.479 | No | 0.0809 |
|  |  |  |  |
| 660 |  |  |  |
| 3mo C57blk6J vs. 6mo C57blk6J | 8.851 | Yes | 0.0004 |
| 3mo C57blk6J vs. 12mo C57blk6J | 4.057 | No | 0.1139 |
| 6mo C57blk6J vs. 12mo C57blk6J | -4.794 | No | 0.0618 |
|  |  |  |  |
| 665 |  |  |  |
| 3mo C57blk6J vs. 6mo C57blk6J | 10.77 | Yes | <0.0001 |
| 3mo C57blk6J vs. 12mo C57blk6J | 7.451 | Yes | 0.0037 |
| 6mo C57blk6J vs. 12mo C57blk6J | -3.315 | No | 0.1963 |
|  |  |  |  |
| 670 |  |  |  |
| 3mo C57blk6J vs. 6mo C57blk6J | 9.631 | Yes | 0.0001 |
| 3mo C57blk6J vs. 12mo C57blk6J | 8.239 | Yes | 0.0014 |
| 6mo C57blk6J vs. 12mo C57blk6J | -1.392 | No | 0.5874 |
|  |  |  |  |
| *675* |  |  |  |
| 3mo C57blk6J vs. 6mo C57blk6J | 7.330 | Yes | 0.0033 |
| 3mo C57blk6J vs. 12mo C57blk6J | 7.176 | Yes | 0.0052 |
| 6mo C57blk6J vs. 12mo C57blk6J | -0.1547 | No | 0.9519 |
|  |  |  |  |
| *680* |  |  |  |
| 3mo C57blk6J vs. 6mo C57blk6J | 4.123 | No | 0.0977 |
| 3mo C57blk6J vs. 12mo C57blk6J | 5.177 | No | 0.0437 |
| 6mo C57blk6J vs. 12mo C57blk6J | 1.054 | No | 0.6812 |

| 1. **residual** | SS | DF | MS | F (DFn, DFd) | P-value |
| --- | --- | --- | --- | --- | --- |
| Wavelength x Age | 19351 | 92 | 210.3 | F (92, 1058) = 11.63 | P<0.0001 |
| Wavelength | 24344 | 46 | 529.2 | F (46, 1058) = 29.26 | P<0.0001 |
| Age | 33948 | 2 | 16974 | F (2, 23) = 35.61 | P<0.0001 |
| Subject | 10963 | 23 | 476.6 | F (23, 1058) = 26.36 | P<0.0001 |
| Residual | 19134 | 1058 | 18.09 |  |  |

| Two-stage linear step-up procedure of Benjamini, Krieger and Yekutieli | Predicted (LS) mean diff. | Discovery | P-Value |
| --- | --- | --- | --- |
|  |  |  |  |
| 450 |  |  |  |
| 3mo C57blk6J vs. 6mo C57blk6J | 2.072 | No | 0.4051 |
| 3mo C57blk6J vs. 12mo C57blk6J | 14.46 | Yes | <0.0001 |
| 6mo C57blk6J vs. 12mo C57blk6J | 12.39 | Yes | <0.0001 |
|  |  |  |  |
| 455 |  |  |  |
| 3mo C57blk6J vs. 6mo C57blk6J | 0.8178 | No | 0.7424 |
| 3mo C57blk6J vs. 12mo C57blk6J | 15.07 | Yes | <0.0001 |
| 6mo C57blk6J vs. 12mo C57blk6J | 14.25 | Yes | <0.0001 |
|  |  |  |  |
| 460 |  |  |  |
| 3mo C57blk6J vs. 6mo C57blk6J | 5.591 | Yes | 0.0248 |
| 3mo C57blk6J vs. 12mo C57blk6J | 20.93 | Yes | <0.0001 |
| 6mo C57blk6J vs. 12mo C57blk6J | 15.34 | Yes | <0.0001 |
|  |  |  |  |
| 465 |  |  |  |
| 3mo C57blk6J vs. 6mo C57blk6J | 12.85 | Yes | <0.0001 |
| 3mo C57blk6J vs. 12mo C57blk6J | 27.63 | Yes | <0.0001 |
| 6mo C57blk6J vs. 12mo C57blk6J | 14.78 | Yes | <0.0001 |
|  |  |  |  |
| 470 |  |  |  |
| 3mo C57blk6J vs. 6mo C57blk6J | 14.83 | Yes | <0.0001 |
| 3mo C57blk6J vs. 12mo C57blk6J | 29.15 | Yes | <0.0001 |
| 6mo C57blk6J vs. 12mo C57blk6J | 14.32 | Yes | <0.0001 |
|  |  |  |  |
| 475 |  |  |  |
| 3mo C57blk6J vs. 6mo C57blk6J | 9.386 | Yes | 0.0002 |
| 3mo C57blk6J vs. 12mo C57blk6J | 23.89 | Yes | <0.0001 |
| 6mo C57blk6J vs. 12mo C57blk6J | 14.50 | Yes | <0.0001 |
|  |  |  |  |
| 480 |  |  |  |
| 3mo C57blk6J vs. 6mo C57blk6J | 1.248 | No | 0.6160 |
| 3mo C57blk6J vs. 12mo C57blk6J | 17.37 | Yes | <0.0001 |
| 6mo C57blk6J vs. 12mo C57blk6J | 16.13 | Yes | <0.0001 |
|  |  |  |  |
| 485 |  |  |  |
| 3mo C57blk6J vs. 6mo C57blk6J | 0.09790 | No | 0.9686 |
| 3mo C57blk6J vs. 12mo C57blk6J | 17.18 | Yes | <0.0001 |
| 6mo C57blk6J vs. 12mo C57blk6J | 17.08 | Yes | <0.0001 |
|  |  |  |  |
| 490 |  |  |  |
| 3mo C57blk6J vs. 6mo C57blk6J | 6.167 | Yes | 0.0133 |
| 3mo C57blk6J vs. 12mo C57blk6J | 21.61 | Yes | <0.0001 |
| 6mo C57blk6J vs. 12mo C57blk6J | 15.44 | Yes | <0.0001 |
|  |  |  |  |
| 495 |  |  |  |
| 3mo C57blk6J vs. 6mo C57blk6J | 13.16 | Yes | <0.0001 |
| 3mo C57blk6J vs. 12mo C57blk6J | 27.27 | Yes | <0.0001 |
| 6mo C57blk6J vs. 12mo C57blk6J | 14.11 | Yes | <0.0001 |
|  |  |  |  |
| 500 |  |  |  |
| 3mo C57blk6J vs. 6mo C57blk6J | 14.44 | Yes | <0.0001 |
| 3mo C57blk6J vs. 12mo C57blk6J | 26.72 | Yes | <0.0001 |
| 6mo C57blk6J vs. 12mo C57blk6J | 12.28 | Yes | <0.0001 |
|  |  |  |  |
| 505 |  |  |  |
| 3mo C57blk6J vs. 6mo C57blk6J | 9.264 | Yes | 0.0002 |
| 3mo C57blk6J vs. 12mo C57blk6J | 21.65 | Yes | <0.0001 |
| 6mo C57blk6J vs. 12mo C57blk6J | 12.39 | Yes | <0.0001 |
|  |  |  |  |
| 510 |  |  |  |
| 3mo C57blk6J vs. 6mo C57blk6J | 2.014 | No | 0.4183 |
| 3mo C57blk6J vs. 12mo C57blk6J | 15.86 | Yes | <0.0001 |
| 6mo C57blk6J vs. 12mo C57blk6J | 13.84 | Yes | <0.0001 |
|  |  |  |  |
| 515 |  |  |  |
| 3mo C57blk6J vs. 6mo C57blk6J | -1.566 | No | 0.5292 |
| 3mo C57blk6J vs. 12mo C57blk6J | 12.53 | Yes | <0.0001 |
| 6mo C57blk6J vs. 12mo C57blk6J | 14.09 | Yes | <0.0001 |
|  |  |  |  |
| 520 |  |  |  |
| 3mo C57blk6J vs. 6mo C57blk6J | 1.171 | No | 0.6379 |
| 3mo C57blk6J vs. 12mo C57blk6J | 13.86 | Yes | <0.0001 |
| 6mo C57blk6J vs. 12mo C57blk6J | 12.69 | Yes | <0.0001 |
|  |  |  |  |
| 525 |  |  |  |
| 3mo C57blk6J vs. 6mo C57blk6J | 8.124 | Yes | 0.0011 |
| 3mo C57blk6J vs. 12mo C57blk6J | 19.49 | Yes | <0.0001 |
| 6mo C57blk6J vs. 12mo C57blk6J | 11.37 | Yes | <0.0001 |
|  |  |  |  |
| 530 |  |  |  |
| 3mo C57blk6J vs. 6mo C57blk6J | 13.80 | Yes | <0.0001 |
| 3mo C57blk6J vs. 12mo C57blk6J | 23.12 | Yes | <0.0001 |
| 6mo C57blk6J vs. 12mo C57blk6J | 9.315 | Yes | 0.0003 |
|  |  |  |  |
| 535 |  |  |  |
| 3mo C57blk6J vs. 6mo C57blk6J | 13.36 | Yes | <0.0001 |
| 3mo C57blk6J vs. 12mo C57blk6J | 23.12 | Yes | <0.0001 |
| 6mo C57blk6J vs. 12mo C57blk6J | 9.759 | Yes | 0.0001 |
|  |  |  |  |
| 540 |  |  |  |
| 3mo C57blk6J vs. 6mo C57blk6J | 8.182 | Yes | 0.0010 |
| 3mo C57blk6J vs. 12mo C57blk6J | 18.36 | Yes | <0.0001 |
| 6mo C57blk6J vs. 12mo C57blk6J | 10.18 | Yes | <0.0001 |
|  |  |  |  |
| 545 |  |  |  |
| 3mo C57blk6J vs. 6mo C57blk6J | 2.223 | No | 0.3717 |
| 3mo C57blk6J vs. 12mo C57blk6J | 13.43 | Yes | <0.0001 |
| 6mo C57blk6J vs. 12mo C57blk6J | 11.21 | Yes | <0.0001 |
|  |  |  |  |
| 550 |  |  |  |
| 3mo C57blk6J vs. 6mo C57blk6J | -2.748 | No | 0.2695 |
| 3mo C57blk6J vs. 12mo C57blk6J | 8.329 | Yes | 0.0012 |
| 6mo C57blk6J vs. 12mo C57blk6J | 11.08 | Yes | <0.0001 |
|  |  |  |  |
| 555 |  |  |  |
| 3mo C57blk6J vs. 6mo C57blk6J | -2.712 | No | 0.2758 |
| 3mo C57blk6J vs. 12mo C57blk6J | 7.980 | Yes | 0.0019 |
| 6mo C57blk6J vs. 12mo C57blk6J | 10.69 | Yes | <0.0001 |
|  |  |  |  |
| 560 |  |  |  |
| 3mo C57blk6J vs. 6mo C57blk6J | 2.318 | No | 0.3516 |
| 3mo C57blk6J vs. 12mo C57blk6J | 11.66 | Yes | <0.0001 |
| 6mo C57blk6J vs. 12mo C57blk6J | 9.345 | Yes | 0.0003 |
|  |  |  |  |
| 565 |  |  |  |
| 3mo C57blk6J vs. 6mo C57blk6J | 9.601 | Yes | 0.0001 |
| 3mo C57blk6J vs. 12mo C57blk6J | 16.32 | Yes | <0.0001 |
| 6mo C57blk6J vs. 12mo C57blk6J | 6.718 | Yes | 0.0089 |
|  |  |  |  |
| 570 |  |  |  |
| 3mo C57blk6J vs. 6mo C57blk6J | 13.42 | Yes | <0.0001 |
| 3mo C57blk6J vs. 12mo C57blk6J | 19.07 | Yes | <0.0001 |
| 6mo C57blk6J vs. 12mo C57blk6J | 5.650 | Yes | 0.0277 |
|  |  |  |  |
| 575 |  |  |  |
| 3mo C57blk6J vs. 6mo C57blk6J | 11.54 | Yes | <0.0001 |
| 3mo C57blk6J vs. 12mo C57blk6J | 17.80 | Yes | <0.0001 |
| 6mo C57blk6J vs. 12mo C57blk6J | 6.256 | Yes | 0.0148 |
|  |  |  |  |
| 580 |  |  |  |
| 3mo C57blk6J vs. 6mo C57blk6J | 7.417 | Yes | 0.0029 |
| 3mo C57blk6J vs. 12mo C57blk6J | 14.73 | Yes | <0.0001 |
| 6mo C57blk6J vs. 12mo C57blk6J | 7.312 | Yes | 0.0044 |
|  |  |  |  |
| 585 |  |  |  |
| 3mo C57blk6J vs. 6mo C57blk6J | 2.360 | No | 0.3429 |
| 3mo C57blk6J vs. 12mo C57blk6J | 9.889 | Yes | 0.0001 |
| 6mo C57blk6J vs. 12mo C57blk6J | 7.529 | Yes | 0.0034 |
|  |  |  |  |
| 590 |  |  |  |
| 3mo C57blk6J vs. 6mo C57blk6J | -2.143 | No | 0.3891 |
| 3mo C57blk6J vs. 12mo C57blk6J | 5.399 | Yes | 0.0355 |
| 6mo C57blk6J vs. 12mo C57blk6J | 7.542 | Yes | 0.0033 |
|  |  |  |  |
| 595 |  |  |  |
| 3mo C57blk6J vs. 6mo C57blk6J | -3.442 | No | 0.1667 |
| 3mo C57blk6J vs. 12mo C57blk6J | 2.754 | No | 0.2830 |
| 6mo C57blk6J vs. 12mo C57blk6J | 6.196 | Yes | 0.0158 |
|  |  |  |  |
| 600 |  |  |  |
| 3mo C57blk6J vs. 6mo C57blk6J | -0.03413 | No | 0.9891 |
| 3mo C57blk6J vs. 12mo C57blk6J | 3.743 | No | 0.1446 |
| 6mo C57blk6J vs. 12mo C57blk6J | 3.778 | No | 0.1410 |
|  |  |  |  |
| 605 |  |  |  |
| 3mo C57blk6J vs. 6mo C57blk6J | 5.303 | Yes | 0.0332 |
| 3mo C57blk6J vs. 12mo C57blk6J | 7.801 | Yes | 0.0024 |
| 6mo C57blk6J vs. 12mo C57blk6J | 2.498 | No | 0.3301 |
|  |  |  |  |
| 610 |  |  |  |
| 3mo C57blk6J vs. 6mo C57blk6J | 11.88 | Yes | <0.0001 |
| 3mo C57blk6J vs. 12mo C57blk6J | 13.89 | Yes | <0.0001 |
| 6mo C57blk6J vs. 12mo C57blk6J | 2.004 | No | 0.4346 |
|  |  |  |  |
| 615 |  |  |  |
| 3mo C57blk6J vs. 6mo C57blk6J | 13.62 | Yes | <0.0001 |
| 3mo C57blk6J vs. 12mo C57blk6J | 15.83 | Yes | <0.0001 |
| 6mo C57blk6J vs. 12mo C57blk6J | 2.212 | No | 0.3884 |
|  |  |  |  |
| 620 |  |  |  |
| 3mo C57blk6J vs. 6mo C57blk6J | 11.38 | Yes | <0.0001 |
| 3mo C57blk6J vs. 12mo C57blk6J | 14.83 | Yes | <0.0001 |
| 6mo C57blk6J vs. 12mo C57blk6J | 3.451 | No | 0.1786 |
|  |  |  |  |
| 625 |  |  |  |
| 3mo C57blk6J vs. 6mo C57blk6J | 7.546 | Yes | 0.0025 |
| 3mo C57blk6J vs. 12mo C57blk6J | 11.74 | Yes | <0.0001 |
| 6mo C57blk6J vs. 12mo C57blk6J | 4.194 | Yes | 0.1022 |
|  |  |  |  |
| 630 |  |  |  |
| 3mo C57blk6J vs. 6mo C57blk6J | 2.824 | No | 0.2566 |
| 3mo C57blk6J vs. 12mo C57blk6J | 7.643 | Yes | 0.0029 |
| 6mo C57blk6J vs. 12mo C57blk6J | 4.819 | No | 0.0604 |
|  |  |  |  |
| 635 |  |  |  |
| 3mo C57blk6J vs. 6mo C57blk6J | -2.108 | No | 0.3968 |
| 3mo C57blk6J vs. 12mo C57blk6J | 2.123 | No | 0.4078 |
| 6mo C57blk6J vs. 12mo C57blk6J | 4.232 | No | 0.0991 |
|  |  |  |  |
| 640 |  |  |  |
| 3mo C57blk6J vs. 6mo C57blk6J | -5.504 | No | 0.0271 |
| 3mo C57blk6J vs. 12mo C57blk6J | -2.793 | No | 0.2762 |
| 6mo C57blk6J vs. 12mo C57blk6J | 2.711 | No | 0.2907 |
|  |  |  |  |
| 645 |  |  |  |
| 3mo C57blk6J vs. 6mo C57blk6J | -6.151 | Yes | 0.0136 |
| 3mo C57blk6J vs. 12mo C57blk6J | -5.506 | Yes | 0.0320 |
| 6mo C57blk6J vs. 12mo C57blk6J | 0.6449 | No | 0.8015 |
|  |  |  |  |
| 650 |  |  |  |
| 3mo C57blk6J vs. 6mo C57blk6J | -1.945 | No | 0.4344 |
| 3mo C57blk6J vs. 12mo C57blk6J | -5.078 | No | 0.0479 |
| 6mo C57blk6J vs. 12mo C57blk6J | -3.133 | No | 0.2220 |
|  |  |  |  |
| 655 |  |  |  |
| 3mo C57blk6J vs. 6mo C57blk6J | 4.034 | No | 0.1052 |
| 3mo C57blk6J vs. 12mo C57blk6J | -0.4454 | No | 0.8621 |
| 6mo C57blk6J vs. 12mo C57blk6J | -4.479 | No | 0.0809 |
|  |  |  |  |
| 660 |  |  |  |
| 3mo C57blk6J vs. 6mo C57blk6J | 8.851 | Yes | 0.0004 |
| 3mo C57blk6J vs. 12mo C57blk6J | 4.057 | No | 0.1139 |
| 6mo C57blk6J vs. 12mo C57blk6J | -4.794 | No | 0.0618 |
|  |  |  |  |
| 665 |  |  |  |
| 3mo C57blk6J vs. 6mo C57blk6J | 10.77 | Yes | <0.0001 |
| 3mo C57blk6J vs. 12mo C57blk6J | 7.451 | Yes | 0.0037 |
| 6mo C57blk6J vs. 12mo C57blk6J | -3.315 | No | 0.1963 |
|  |  |  |  |
| 670 |  |  |  |
| 3mo C57blk6J vs. 6mo C57blk6J | 9.631 | Yes | 0.0001 |
| 3mo C57blk6J vs. 12mo C57blk6J | 8.239 | Yes | 0.0014 |
| 6mo C57blk6J vs. 12mo C57blk6J | -1.392 | No | 0.5874 |
|  |  |  |  |
| *675* |  |  |  |
| 3mo C57blk6J vs. 6mo C57blk6J | 7.330 | Yes | 0.0033 |
| 3mo C57blk6J vs. 12mo C57blk6J | 7.176 | Yes | 0.0052 |
| 6mo C57blk6J vs. 12mo C57blk6J | -0.1547 | No | 0.9519 |
|  |  |  |  |
| *680* |  |  |  |
| 3mo C57blk6J vs. 6mo C57blk6J | 4.123 | No | 0.0977 |
| 3mo C57blk6J vs. 12mo C57blk6J | 5.177 | No | 0.0437 |
| 6mo C57blk6J vs. 12mo C57blk6J | 1.053 | No | 0.6812 |

**Supplementary Table 7. Statistics for B6C3H mice**

Two-way repeated measures ANOVA where, DF (degrees of freedom), SS (sum of squares), MS (mean square), and post-hoc comparisons of normal ageing in B6C3H mice **(A)** % reflectance, and **(B)** residual

| 1. **% reflectance** | SS | DF | MS | F (DFn, DFd) | P-value |
| --- | --- | --- | --- | --- | --- |
| Wavelength x Age | 4386 | 46 | 95.34 | F (46, 1196) = 9.695 | P<0.0001 |
| Wavelength | 169768 | 46 | 3691 | F (46, 1196) = 375.3 | P<0.0001 |
| Age | 1230 | 1 | 1230 | F (1, 26) = 3.010 | P=0.0946 |
| Subject | 10625 | 26 | 408.6 | F (26, 1196) = 41.55 | P<0.0001 |
| Residual | 11762 | 1196 | 9.834 |  |  |

| Two-stage linear step-up procedure of Benjamini, Krieger and Yekutieli | Predicted (LS) mean diff. | Discovery | P- Value |
| --- | --- | --- | --- |
| 450 | 2.352 | No | 0.1557 |
| 455 | 5.255 | Yes | 0.0015 |
| 460 | 6.249 | Yes | 0.0002 |
| 465 | 4.156 | Yes | 0.0122 |
| 470 | 1.219 | No | 0.4617 |
| 475 | 0.4368 | No | 0.7920 |
| 480 | 3.305 | No | 0.0462 |
| 485 | 6.055 | Yes | 0.0003 |
| 490 | 6.066 | Yes | 0.0003 |
| 495 | 2.702 | No | 0.1031 |
| 500 | -0.3309 | No | 0.8417 |
| 505 | -1.273 | No | 0.4421 |
| 510 | 0.2732 | No | 0.8690 |
| 515 | 3.008 | No | 0.0696 |
| 520 | 5.980 | Yes | 0.0003 |
| 525 | 5.689 | Yes | 0.0006 |
| 530 | 3.849 | Yes | 0.0203 |
| 535 | 1.120 | No | 0.4992 |
| 540 | -0.7177 | No | 0.6648 |
| 545 | -0.9153 | No | 0.5806 |
| 550 | 1.286 | No | 0.4374 |
| 555 | 4.741 | Yes | 0.0043 |
| 560 | 7.257 | Yes | <0.0001 |
| 565 | 7.100 | Yes | <0.0001 |
| 570 | 4.646 | Yes | 0.0051 |
| 575 | 1.478 | No | 0.3723 |
| 580 | -0.8513 | No | 0.6073 |
| 585 | -0.9094 | No | 0.5830 |
| 590 | 0.6061 | No | 0.7145 |
| 595 | 3.907 | Yes | 0.0185 |
| 600 | 7.344 | Yes | <0.0001 |
| 605 | 8.518 | Yes | <0.0001 |
| 610 | 6.486 | Yes | <0.0001 |
| 615 | 3.486 | Yes | 0.0355 |
| 620 | 0.2753 | No | 0.8680 |
| 625 | -2.605 | No | 0.1161 |
| 630 | -4.621 | Yes | 0.0053 |
| 635 | -4.969 | Yes | 0.0028 |
| 640 | -2.417 | No | 0.1446 |
| 645 | 1.287 | No | 0.4373 |
| 650 | 3.238 | No | 0.0508 |
| 655 | 4.172 | Yes | 0.0119 |
| 660 | 3.311 | No | 0.0458 |
| 665 | 1.581 | No | 0.3398 |
| 670 | -1.463 | No | 0.3771 |
| 675 | -5.409 | Yes | 0.0011 |
| 680 | -8.920 | Yes | <0.0001 |

| 1. **residual** | SS | DF | MS | F (DFn, DFd) | P-value |
| --- | --- | --- | --- | --- | --- |
| Wavelength x Genotype | 4386 | 46 | 95.34 | F (46, 1196) = 9.695 | P<0.0001 |
| Wavelength | 4386 | 46 | 95.34 | F (46, 1196) = 9.695 | P<0.0001 |
| Genotype | 1230 | 1 | 1230 | F (1, 26) = 3.010 | P=0.0946 |
| Subject | 10624 | 26 | 408.6 | F (26, 1196) = 41.55 | P<0.0001 |
| Residual | 11762 | 1196 | 9.834 |  |  |

| Two-stage linear step-up procedure of Benjamini, Krieger and Yekutieli | Predicted (LS) mean diff. | Discovery | P-Value |
| --- | --- | --- | --- |
| 450 | 2.352 | No | 0.1557 |
| 455 | 5.255 | Yes | 0.0015 |
| 460 | 6.248 | Yes | 0.0002 |
| 465 | 4.156 | Yes | 0.0122 |
| 470 | 1.219 | No | 0.4617 |
| 475 | 0.4368 | No | 0.7920 |
| 480 | 3.305 | No | 0.0462 |
| 485 | 6.055 | Yes | 0.0003 |
| 490 | 6.066 | Yes | 0.0003 |
| 495 | 2.702 | No | 0.1031 |
| 500 | -0.3309 | No | 0.8417 |
| 505 | -1.273 | No | 0.4421 |
| 510 | 0.2732 | No | 0.8690 |
| 515 | 3.008 | No | 0.0696 |
| 520 | 5.980 | Yes | 0.0003 |
| 525 | 5.689 | Yes | 0.0006 |
| 530 | 3.849 | Yes | 0.0203 |
| 535 | 1.120 | No | 0.4992 |
| 540 | -0.7177 | No | 0.6648 |
| 545 | -0.9153 | No | 0.5806 |
| 550 | 1.286 | No | 0.4375 |
| 555 | 4.741 | Yes | 0.0043 |
| 560 | 7.257 | Yes | <0.0001 |
| 565 | 7.100 | Yes | <0.0001 |
| 570 | 4.646 | Yes | 0.0051 |
| 575 | 1.478 | No | 0.3723 |
| 580 | -0.8513 | No | 0.6073 |
| 585 | -0.9094 | No | 0.5830 |
| 590 | 0.6061 | No | 0.7145 |
| 595 | 3.907 | Yes | 0.0185 |
| 600 | 7.344 | Yes | <0.0001 |
| 605 | 8.518 | Yes | <0.0001 |
| 610 | 6.486 | Yes | <0.0001 |
| 615 | 3.486 | Yes | 0.0355 |
| 620 | 0.2753 | No | 0.8680 |
| 625 | -2.605 | No | 0.1161 |
| 630 | -4.621 | Yes | 0.0053 |
| 635 | -4.969 | Yes | 0.0028 |
| 640 | -2.417 | No | 0.1446 |
| 645 | 1.287 | No | 0.4373 |
| 650 | 3.238 | No | 0.0508 |
| 655 | 4.172 | Yes | 0.0119 |
| 660 | 3.311 | No | 0.0458 |
| 665 | 1.581 | No | 0.3398 |
| 670 | -1.463 | No | 0.3771 |
| 675 | -5.409 | Yes | 0.0011 |
| 680 | -8.920 | Yes | <0.0001 |

**Supplementary Table 8. Statistics for Sv129B mice**

Two-way repeated measures ANOVA where, DF (degrees of freedom), SS (sum of squares), MS (mean square), and post-hoc comparisons of normal ageing in Sv129B mice **(A)** % reflectance, and **(B)** residual

| 1. **% reflectance** | SS | DF | MS | F (DFn, DFd) | P-value |
| --- | --- | --- | --- | --- | --- |
| Wavelength x Genotype | 3282 | 46 | 71.35 | F (46, 1012) = 10.61 | P<0.0001 |
| Wavelength | 205776 | 46 | 4473 | F (46, 1012) = 665.3 | P<0.0001 |
| Genotype | 1861 | 1 | 1861 | F (1, 22) = 5.826 | P=0.0246 |
| Subject | 7027 | 22 | 319.4 | F (22, 1012) = 47.51 | P<0.0001 |
| Residual | 6804 | 1012 | 6.724 |  |  |

| Two-stage linear step-up procedure of Benjamini, Krieger and Yekutieli | Predicted (LS) mean diff. | Discovery | P-Value |
| --- | --- | --- | --- |
| 8mo Sv129B - 18mo Sv129B |  |  |  |
| 450 | 4.728 | Yes | 0.0018 |
| 455 | 4.501 | Yes | 0.0030 |
| 460 | 3.651 | Yes | 0.0161 |
| 465 | 5.003 | Yes | 0.0010 |
| 470 | 7.734 | Yes | <0.0001 |
| 475 | 9.058 | Yes | <0.0001 |
| 480 | 7.716 | Yes | <0.0001 |
| 485 | 6.646 | Yes | <0.0001 |
| 490 | 5.296 | Yes | 0.0005 |
| 495 | 4.189 | Yes | 0.0058 |
| 500 | 5.131 | Yes | 0.0007 |
| 505 | 7.064 | Yes | <0.0001 |
| 510 | 7.656 | Yes | <0.0001 |
| 515 | 6.589 | Yes | <0.0001 |
| 520 | 4.772 | Yes | 0.0017 |
| 525 | 3.105 | Yes | 0.0406 |
| 530 | 2.905 | Yes | 0.0553 |
| 535 | 3.168 | Yes | 0.0367 |
| 540 | 4.176 | Yes | 0.0059 |
| 545 | 5.294 | Yes | 0.0005 |
| 550 | 4.627 | Yes | 0.0023 |
| 555 | 4.278 | Yes | 0.0048 |
| 560 | 3.441 | Yes | 0.0233 |
| 565 | 2.029 | No | 0.1805 |
| 570 | 2.392 | No | 0.1146 |
| 575 | 2.906 | Yes | 0.0552 |
| 580 | 3.610 | Yes | 0.0173 |
| 585 | 3.454 | Yes | 0.0228 |
| 590 | 3.742 | Yes | 0.0136 |
| 595 | 2.150 | No | 0.1560 |
| 600 | 0.9634 | No | 0.5248 |
| 605 | -0.2275 | No | 0.8806 |
| 610 | -1.441 | No | 0.3415 |
| 615 | -0.6597 | No | 0.6632 |
| 620 | 0.5145 | No | 0.7341 |
| 625 | 0.1247 | No | 0.9344 |
| 630 | 1.493 | No | 0.3245 |
| 635 | 2.738 | Yes | 0.0709 |
| 640 | 1.777 | No | 0.2409 |
| 645 | 0.3831 | No | 0.8003 |
| 650 | -1.287 | No | 0.3955 |
| 655 | -2.210 | No | 0.1448 |
| 660 | -2.893 | Yes | 0.0563 |
| 665 | -4.621 | Yes | 0.0023 |
| 670 | -6.305 | Yes | <0.0001 |
| 675 | -5.000 | Yes | 0.0010 |
| 680 | -1.911 | No | 0.2071 |

| 1. **residual** | SS | DF | MS | F (DFn, DFd) | P-value |
| --- | --- | --- | --- | --- | --- |
| Wavelength x Age | 3282 | 46 | 71.35 | F (46, 1012) = 10.61 | P<0.0001 |
| Wavelength | 3282 | 46 | 71.35 | F (46, 1012) = 10.61 | P<0.0001 |
| Age | 1861 | 1 | 1861 | F (1, 22) = 5.826 | P=0.0246 |
| Subject | 7027 | 22 | 319.4 | F (22, 1012) = 47.51 | P<0.0001 |
| Residual | 6804 | 1012 | 6.724 |  |  |

| Two-stage linear step-up procedure of Benjamini, Krieger and Yekutieli | Predicted (LS) mean diff. | Discovery | P-Value |
| --- | --- | --- | --- |
| 450 | 4.728 | Yes | 0.0018 |
| 455 | 4.501 | Yes | 0.0030 |
| 460 | 3.651 | Yes | 0.0161 |
| 465 | 5.003 | Yes | 0.0010 |
| 470 | 7.734 | Yes | <0.0001 |
| 475 | 9.058 | Yes | <0.0001 |
| 480 | 7.716 | Yes | <0.0001 |
| 485 | 6.646 | Yes | <0.0001 |
| 490 | 5.296 | Yes | 0.0005 |
| 495 | 4.189 | Yes | 0.0058 |
| 500 | 5.131 | Yes | 0.0007 |
| 505 | 7.064 | Yes | <0.0001 |
| 510 | 7.656 | Yes | <0.0001 |
| 515 | 6.589 | Yes | <0.0001 |
| 520 | 4.772 | Yes | 0.0017 |
| 525 | 3.105 | Yes | 0.0406 |
| 530 | 2.905 | Yes | 0.0553 |
| 535 | 3.168 | Yes | 0.0367 |
| 540 | 4.176 | Yes | 0.0059 |
| 545 | 5.294 | Yes | 0.0005 |
| 550 | 4.627 | Yes | 0.0023 |
| 555 | 4.278 | Yes | 0.0048 |
| 560 | 3.441 | Yes | 0.0233 |
| 565 | 2.029 | No | 0.1805 |
| 570 | 2.392 | No | 0.1146 |
| 575 | 2.906 | Yes | 0.0552 |
| 580 | 3.610 | Yes | 0.0173 |
| 585 | 3.454 | Yes | 0.0228 |
| 590 | 3.742 | Yes | 0.0136 |
| 595 | 2.150 | No | 0.1560 |
| 600 | 0.9634 | No | 0.5248 |
| 605 | -0.2275 | No | 0.8806 |
| 610 | -1.441 | No | 0.3415 |
| 615 | -0.6597 | No | 0.6632 |
| 620 | 0.5145 | No | 0.7341 |
| 625 | 0.1247 | No | 0.9344 |
| 630 | 1.493 | No | 0.3245 |
| 635 | 2.738 | Yes | 0.0709 |
| 640 | 1.777 | No | 0.2409 |
| 645 | 0.3831 | No | 0.8003 |
| 650 | -1.287 | No | 0.3955 |
| 655 | -2.210 | No | 0.1448 |
| 660 | -2.893 | Yes | 0.0563 |
| 665 | -4.621 | Yes | 0.0023 |
| 670 | -6.305 | Yes | <0.0001 |
| 675 | -5.000 | Yes | 0.0010 |
| 680 | -1.911 | No | 0.2071 |
